# Minocycline-induced disruption of the intestinal FXR/FGF15 axis impairs osteogenesis in mice

**DOI:** 10.1172/jci.insight.160578

**Published:** 2023-01-10

**Authors:** Matthew D. Carson, Amy J. Warner, Jessica D. Hathaway-Schrader, Vincenza L. Geiser, Joseph Kim, Joy E. Gerasco, William D. Hill, John J. Lemasters, Alexander V. Alekseyenko, Yongren Wu, Hai Yao, J. Ignacio Aguirre, Caroline Westwater, Chad M. Novince

**Affiliations:** 1 Department of Oral Health Sciences, College of Dental Medicine;; 2 Department of Pediatrics, Division of Endocrinology, College of Medicine,; 3 Department of Stomatology, Division of Periodontics, College of Dental Medicine;; 4 Department of Drug Discovery & Biomedical Sciences, College of Pharmacy;; 5 Department of Pathology and Laboratory Medicine, College of Medicine;; 6 Department of Biochemistry & Molecular Biology, College of Medicine;; 7 Biomedical Informatics Center, Program for Human Microbiome Research, Department of Public Health Sciences, College of Medicine;; 8 Department of Healthcare Leadership and Management, College of Health Professions; and; 9 Department of Orthopedics & Physical Medicine, College of Medicine, Medical University of South Carolina, Charleston, South Carolina, USA.; 10 Department of Bioengineering, College of Engineering, Clemson University, Clemson, South Carolina, USA.; 11 Department of Physiological Sciences, College of Veterinary Medicine, University of Florida, Gainesville, Florida, USA.; 12 Department of Microbiology and Immunology, Hollings Cancer Center, Medical University of South Carolina, Charleston, South Carolina, USA.

**Keywords:** Bone Biology, Endocrinology, Osteoclast/osteoblast biology

## Abstract

Antibiotic-induced shifts in the indigenous gut microbiota influence normal skeletal maturation. Current theory implies that gut microbiota actions on bone occur through a direct gut/bone signaling axis. However, our prior work supports that a gut/liver signaling axis contributes to gut microbiota effects on bone. Our purpose was to investigate the effects of minocycline, a systemic antibiotic treatment for adolescent acne, on pubertal/postpubertal skeletal maturation. Sex-matched specific pathogen–free (SPF) and germ-free (GF) C57BL/6T mice were administered a clinically relevant minocycline dose from age 6–12 weeks. Minocycline caused dysbiotic shifts in the gut bacteriome and impaired skeletal maturation in SPF mice but did not alter the skeletal phenotype in GF mice. Minocycline administration in SPF mice disrupted the intestinal farnesoid X receptor/fibroblast growth factor 15 axis, a gut/liver endocrine axis supporting systemic bile acid homeostasis. Minocycline-treated SPF mice had increased serum conjugated bile acids that were farnesoid X receptor (FXR) antagonists, suppressed osteoblast function, decreased bone mass, and impaired bone microarchitecture and fracture resistance. Stimulating osteoblasts with the serum bile acid profile from minocycline-treated SPF mice recapitulated the suppressed osteogenic phenotype found in vivo, which was mediated through attenuated FXR signaling. This work introduces bile acids as a potentially novel mediator of gut/liver signaling actions contributing to gut microbiota effects on bone.

## Graphical abstract

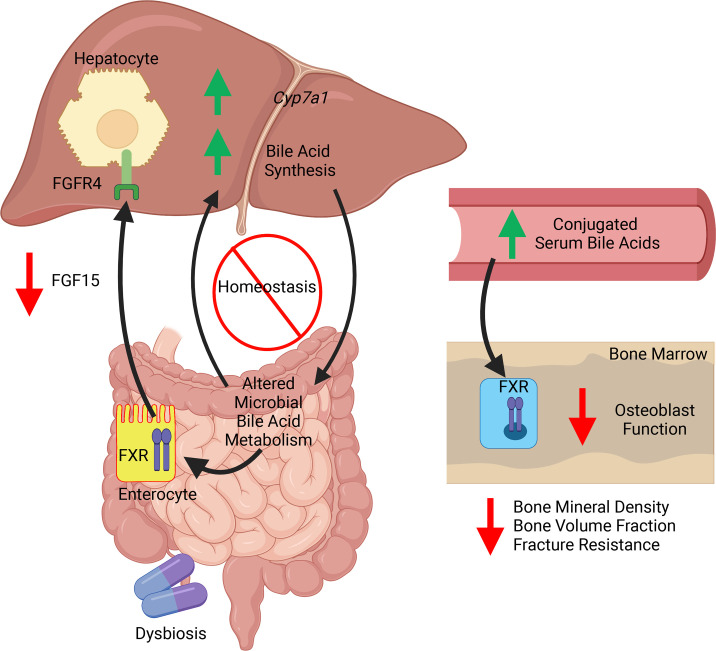

## Introduction

The gut microbiota is the collection of microorganisms colonizing the gastrointestinal tract. Gut microbiota interactions with the host influence the development and homeostasis of host tissues, both locally in the gut and at distant extragastrointestinal sites (1–4). The gut microbiota regulates the actions of bone cells, which affects normal postnatal skeletal development (5–8). We and others have recently shown that the composition of the healthy indigenous gut microbiota critically influences normal skeletal maturation (7, 8).

Antibiotic-induced shifts in the indigenous gut microbiota can cause a dysbiotic relationship between the host and gut microbes, which has detrimental effects on host physiology and metabolism (4, 9–12). The gut microbiota’s resiliency to recover from antibiotic-induced dysbiosis is impaired by antibiotic administration during critical developmental windows and extensive courses of antibiotic therapy (9–12). We have shown previously that antibiotic-induced gut dysbiosis during early life has lasting effects on hepatic metabolism and postnatal skeletal development (13). We have also previously demonstrated that antibiotic-induced gut dysbiosis during the pubertal/postpubertal growth phase has detrimental effects on skeletal maturation (14).

The pubertal/postpubertal growth phase is a critical window of skeletal maturation (15–17). Adolescence, which encompasses the pubertal/postpubertal developmental stage, is a period of rapid growth and maturation between childhood and young adulthood. Clinical studies evaluating the accrual of bone mass across the life span have shown that up to 40%–50% of peak bone mass accrual is realized during adolescence (15–17). Optimizing the attainment of bone mass during adolescence is an important determinant of osteoporosis and fracture risk throughout life (15, 18, 19).

Gut microbiota actions regulating bone cell functions are mediated by microbially derived ligands and metabolites (20, 21). However, the signaling mechanisms are poorly understood. Our published work (7, 22) has challenged the current paradigm that gut microbiota actions on the skeleton occur through a direct signaling axis from gut to bone (20, 21). We previously showed that the gut microbiota modulates liver-derived factors that signal at bone cells, which supported proposing that a gut/liver signaling axis contributes to gut microbiota’s effects on bone metabolism (7, 22). Herein, we introduce bile acids as a potentially novel mediator of gut/liver signaling actions contributing to gut microbiota effects on the skeleton.

The intestinal farnesoid X receptor (FXR)/fibroblast growth factor 15 (FGF15) axis is a gut/liver endocrine axis that suppresses hepatic bile acid synthesis to support systemic bile acid homeostasis (23–29). Primary bile acids are synthesized from cholesterol in the liver, conjugated with taurine or glycine, and excreted into the intestine, where the microbiota metabolizes them (23–29). Bile acids act as agonists or antagonists at the nuclear FXR, which intestinal enterocytes express at high levels (23–29). Bile acid activation of enterocyte/FXR induces the production of intestinal FGF15 (rodent ortholog of human FGF19) (23–29). Intestinal FGF15/19 is a hormone-like factor that signals at hepatocyte/fibroblast growth factor receptor 4 (FGFR4) to suppress cholesterol 7α-hydroxylase–mediated (CYP7A1-mediated) liver bile acid synthesis and support systemic bile acid homeostasis (23–29).

Bozadjieva-Kramer et al. performed vertical gastrectomy-induced osteopenia studies in intestinal specific FGF15-null mice, revealing that intestinal FGF15 has positive effects on skeletal metabolism (30). The authors postulated that intestinal FGF15 protects against bone loss by restricting serum bile acid levels, but the role of the intestinal FXR/FGF15 axis and serum bile acids were not investigated (30). Appreciating that the FXR/FGF15 axis regulates endogenous intestinal FGF15 production, work by Bozadjieva-Kramer et al. supports the concept that the intestinal FXR/FGF15 axis is a candidate regulator of bone metabolism.

Gut microbiota metabolism of intestinal bile acids modulates the intestinal FXR/FGF15 axis and systemic bile acid homeostasis. Intestinal bacteria encode unique enzymes that differentially deconjugate bile acids and transform primary bile acids to secondary bile acids (25, 26, 31, 32). Therefore, changes in the composition of gut bacterial communities alter the microbial biotransformation of intestinal bile acids (25, 26, 31, 32). Of interest, prior reports have shown that drug-induced modifications in gut bacteria composition/function alter the intestinal bile acid pool’s capacity to activate the intestinal FXR/FGF15 axis (33–36).

The purpose of this study was to investigate the effects of minocycline, a common systemic tetracycline antibiotic treatment for adolescent acne, on pubertal/postpubertal skeletal maturation. Acne is the most common skin condition and afflicts about 85% of adolescents and young adults (37–39). Roughly one-third of adolescents and young adults with acne are prescribed systemic antibiotics at antimicrobial doses, and about 40% of these patients are administered minocycline (39–43). The mean duration of systemic antibiotic therapy for acne in adolescents and young adults ranges from 4 to 11 months (39–43). Systemic minocycline therapy has been shown to cause shifts in the healthy gut microbiome of humans and rodents (44–47). However, minocycline effects on bile acid metabolism and skeletal maturation are unknown. This report demonstrates that minocycline-induced gut dysbiosis during pubertal/postpubertal growth suppresses osteoblast function and impairs skeletal maturation. Minocycline-induced detrimental effects on osteogenesis were linked to disruption of the intestinal FXR/FGF15 axis and dysregulated serum bile acids. This work reveals that bile acids are a potentially novel mediator of gut/liver signaling actions contributing to gut microbiota effects on bone metabolism.

## Results

### Study design and timeline.

We treated C57BL/6T mice with a clinically relevant minocycline dose or vehicle control to determine whether antibiotics administered during the pubertal/postpubertal growth phase affect skeletal maturation. The study timeline was designed to model the clinical administration of antibiotics to adolescents for acne treatment. Treatment was initiated at age 6 weeks since C57BL/6 mice typically reach puberty by age 5 to 6 weeks (48). Further, age 6 weeks is the onset of the pubertal/postpubertal phase of skeletal growth and maturation in C57BL/6 mice (49, 50). Experimental groups were euthanized at age 12 weeks, which is when bone modeling/growth is principally complete in C57BL/6 mice (51, 52). Six weeks after antibiotic therapy, experimental groups were euthanized at age 18 weeks, the age when the skeleton is considered mature in C57BL/6 mice (52, 53).

**Figure 1:**
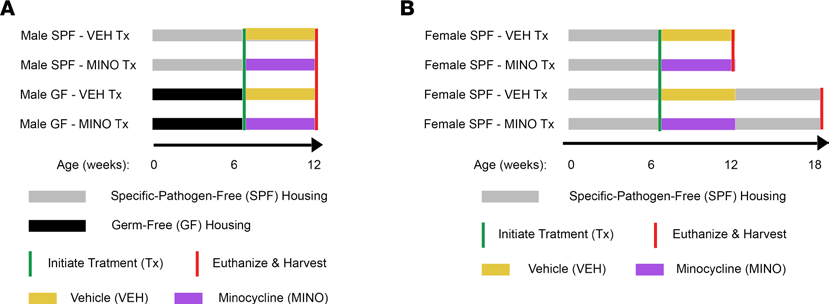
Study design and timeline. (**A**) Male C57BL/6T mice, reared under both specific pathogen–free (SPF) and germ-free (GF) conditions, were treated with minocycline hydrochloride or vehicle control from age 6 to 12 weeks. Male mice were euthanized at age 12 weeks to discern minocycline treatment effects that are microbiota dependent. (**B**) Female C57BL/6T mice, reared under SPF conditions, were treated with minocycline or vehicle from age 6 to 12 weeks. Female mice were euthanized at age 12 and 18 weeks to assess immediate and sustained minocycline treatment effects.

Male C57BL/6T mice were administered minocycline from age 6 to 12 weeks, both under specific pathogen–free (SPF) and germ-free (GF) barrier conditions. We euthanized the male mice at age 12 weeks, to determine whether minocycline effects on skeletal maturation are dependent on the gut microbiota (Figure 1A). In a separate experiment, female C57BL/6T SPF mice were administered minocycline from age 6 to 12 weeks. The female SPF mice were euthanized at ages 12 and 18 weeks, to evaluate sex differences and immediate and sustained antibiotic treatment effects (Figure 1B).

### Minocycline therapy during pubertal/postpubertal growth impairs skeletal maturation and suppresses osteoblastogenesis.

**Figure 2:**
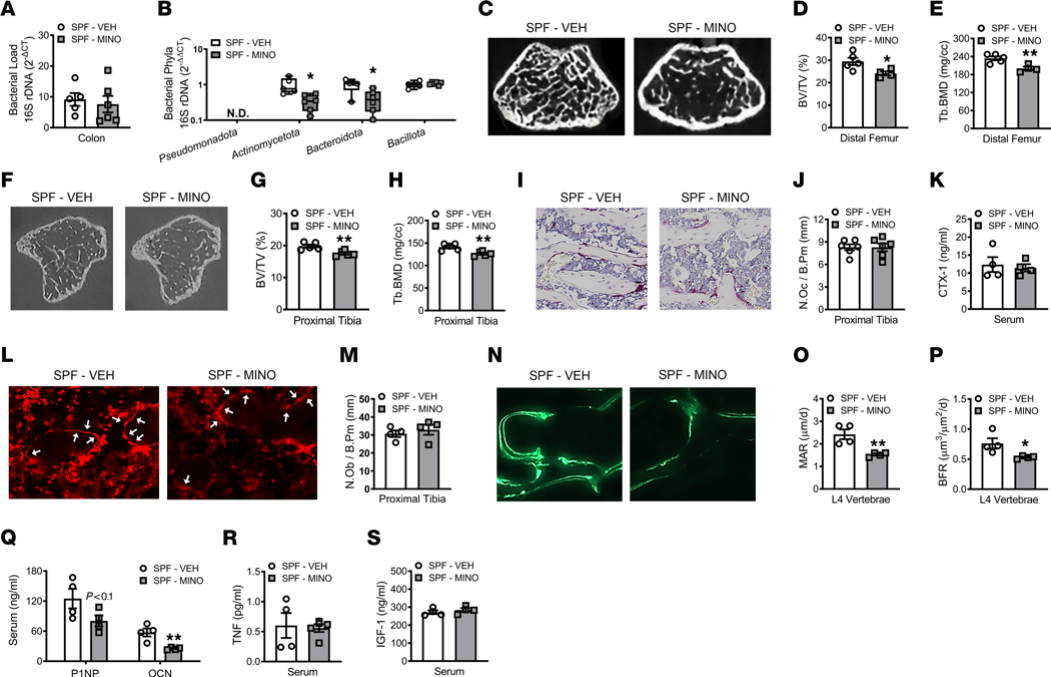
Minocycline therapy during pubertal/postpubertal growth impairs skeletal maturation and suppresses osteoblastogenesis. Male C57BL/6T specific pathogen–free (SPF) mice were administered vehicle control (VEH) or minocycline (MINO) from age 6 to 12 weeks; euthanized at age 12 weeks. Quantitative real-time PCR (qRT-PCR) 16s rDNA analysis of colonic contents evaluating (**A**) bacterial load and (**B**) phyla; *n* = 5–6/group. (**A**) Bacterial load determined by normalizing the universal 16S gene to a bacterial DNA standard; quantification by the 2^-ΔCT^ method. (**B**) Phylum outcomes determined by normalizing phyla genes to the universal 16S gene; quantification via the 2^-ΔΔCT^ method. N.D., not detected. Micro-CT analysis of distal femur trabecular bone; *n* = 4–5/group: (**C**) representative images; (**D**) bone volume per tissue volume (BV/TV); (**E**) trabecular bone mineral density (Tb.BMD). Micro-CT analysis of proximal tibia trabecular bone; *n* = 5/group: (**F**) representative images; (**G**) BV/TV; (**H**) Tb.BMD. Histomorphometric analysis of tartrate-resistant acid phosphatase–positive (TRAP^+^) osteoclasts lining trabecular bone in the proximal tibia; *n* = 6/group: (**I**) representative images (original magnification, 200×); (**J**) number of osteoclasts per bone perimeter (N.Oc/B.Pm). (**K**) C-terminal telopeptides of type I collagen (CTX-I) serum ELISA; *n* = 4/group. Immunofluorescence analysis of osteoblasts lining trabecular bone in the proximal tibia. Osterix^+^ cuboidal bone lining cells were designated osteoblasts (red, osterix–rhodamine); *n* = 4/group: (**L**) representative images (original magnification, 200×), arrows indicate osteoblasts; (**M**) number of osteoblasts per bone perimeter (N.Ob/B.Pm). Dynamic histomorphometric analysis of trabecular bone formation indexes in L4 vertebra; calcein administered 5 and 2 days prior to sacrifice; *n* = 4/group: (**N**) representative images (original magnification, 200×); (**O**) mineral apposition rate (MAR); (**P**) bone formation rate (BFR). (**Q**) N-terminal propeptide of type 1 procollagen (P1NP) and osteocalcin (OCN) serum ELISAs; *n* = 4/group. (**R**) Tumor necrosis factor (TNF) and (**S**) insulin-like growth factor 1 (IGF-1) serum ELISAs; *n* = 4–5/group. Unpaired 2-tailed *t* test; reported as mean ± SEM; *P < 0.05 versus VEH, **P < 0.01 versus VEH.

Treating male SPF mice with a clinically relevant minocycline dose from age 6 to 12 weeks (Figure 1A) did not alter the colonic bacterial load (Figure 2A). However, minocycline caused phylum-level shifts in the colonic bacteriome, with reduced presence of Actinomycetota and Bacteroidota (formerly Actinobacteria and Bacteroidetes) (54) (Figure 2B).

Body weight, body length, and tibia length were similar in minocycline- versus vehicle-treated male SPF mice (Supplemental Figure 1, A–D; supplemental material available online with this article; https://doi.org/10.1172/jci.insight.160578DS1), which implies minocycline did not disrupt normal somatic growth processes. Distal femur (Figure 2, C–E) and proximal tibia (Figure 2, F–H) micro-CT analyses showed that minocycline blunted the trabecular bone volume fraction and bone mineral density in male SPF mice. The inferior trabecular bone volume fraction in the distal femur and the proximal tibia was attributed to a decrease in trabecular number (Supplemental Figure 1, E and H). Micro-CT analyses of cortical bone parameters in the mid-diaphysis of the femur (Supplemental Figure 1, K–N) and tibia (Supplemental Figure 1, O–R) showed that minocycline did not affect cortical bone area fraction, cortical thickness, or cortical bone mineral density in male SPF mice.

Minocycline therapy did not affect TRAP^+^ osteoclast cell numbers lining trabecular bone in the proximal tibia of male SPF mice (Figure 2, I and J). Serum C-terminal telopeptides of type I collagen (CTX-I), a marker for osteoclast resorptive function, was similar in minocycline- versus vehicle-treated male SPF mice (Figure 2K). No differences were found in the number of osterix^+^ osteoblasts lining trabecular bone surfaces of the proximal tibia in minocycline- versus vehicle-treated male SPF mice (Figure 2, L and M). Dynamic histomorphometric analysis in calcein pulse-labeled lumbar vertebrae sections demonstrated minocycline treatment did not alter the mineralized surface per bone surface (Supplemental Figure 1S) but blunted the trabecular mineral apposition rate and bone formation rate (Figure 2, N–P). We validated that minocycline therapy suppresses osteoblast function by assessing serum bone formation markers, N-terminal propeptide of type 1 procollagen (P1NP) and osteocalcin (OCN). Paralleling the blunted mineral apposition and bone formation rates (Figure 2, N–P), serum P1NP and OCN (Figure 2Q) were suppressed in minocycline- versus vehicle-treated male SPF mice. Gut microbiota actions promoting osteoclastogenesis and suppressing osteoblastogenesis have been linked to changes in circulating levels of tumor necrosis factor (TNF) and/or insulin-like growth factor 1 (IGF-1) (5, 6, 14, 22). However, minocycline therapy did not alter serum levels of TNF (Figure 2R) or IGF-1 (Figure 2S). These data reveal that minocycline therapy impairs pubertal/postpubertal skeletal maturation in SPF mice through suppressed osteoblast function, which does not appear to be attributed to altered circulating levels of TNF or IGF-1.

### Minocycline does not alter gastrointestinal immunity or cause cytotoxic effects in kidney or liver.

**Figure 3:**
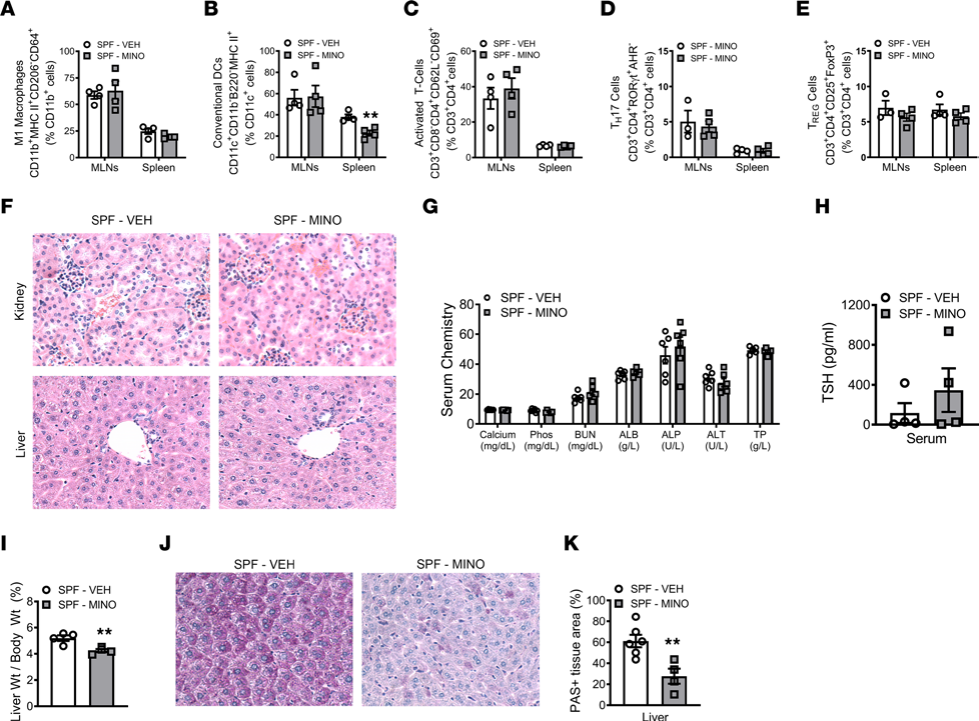
Minocycline does not alter gastrointestinal immunity or cause cytotoxic effects in kidney or liver. Male C57BL/6T specific pathogen–free (SPF) mice were administered vehicle control (VEH) or minocycline (MINO) from age 6 to 12 weeks; euthanized at age 12 weeks. (**A**–**E**) Flow cytometric analysis in spleen and mesenteric lymph node (MLN) cells. (**A**) CD11b^+^MHCII^+^CD206^–^CD64^+^ M1 macrophages, reported as % CD11b^+^ cells; *n* = 4/group. (**B**) CD11c^+^CD11b^–^B220^–^MHCII^+^ conventional dendritic cells (DCs); reported as % CD11c^+^ cells; *n* = 4/group. (**C**) CD3^+^CD8^−^CD4^+^CD62L^−^CD69^+^ activated T cells; reported as % CD3^+^CD4^+^ cells; *n* = 4/group. (**D**) CD3^+^CD4^+^RORγt^+^AHR^−^ T_H_17 cells and (**E**) CD3^+^CD4^+^CD25^+^FoxP3^+^ T_REG_ cells; reported as % CD3^+^CD4^+^ cells; *n* = 3–4/group. (**F**) Representative H&E-stained kidney and liver sections used for histopathological evaluation (original magnification, 200×). (**G**) Serum chemistry analysis: calcium, phosphorus (Phos), blood urea nitrogen (BUN), albumin (ALB), alkaline phosphatase (ALP), alanine aminotransferase (ALT), total protein (TP); *n* = 6/group. (**H**) Thyroid-stimulating hormone (TSH) serum ELISA; *n* = 4/group. (**I**) Liver weight per body weight; *n* = 4/group. Periodic acid–Schiff–stained (PAS-stained) median liver lobe sections; *n* = 4–6/group: (**J**) representative images (original magnification, 200×), (**K**) PAS^+^ area per tissue area (%). Unpaired 2-tailed *t* test; reported as mean ± SEM; **P < 0.01 vs. VEH.

Considering that antibiotic-induced changes in the gut microbiota can dysregulate gastrointestinal and systemic immunity (9–12), we performed flow cytometric analysis in the mesenteric lymph nodes (MLNs) and spleen. The frequency of M1 macrophages was similar in the MLNs and spleen of minocycline- versus vehicle-treated male SPF mice (Figure 3A). Minocycline treatment had no effect on conventional dendritic cells in gut-draining MLNs but reduced conventional dendritic cells in the spleen (Figure 3B). Activated T helper cells (Figure 3C), T_H_17 cells (Figure 3D), and T_REG_ cells (Figure 3E) were not different in the MLNs or spleen of minocycline- versus vehicle-treated male SPF mice. These results demonstrate that minocycline does not induce a proinflammatory response in SPF mice.

Systemic minocycline therapy has been linked with hepatoxicity, nephritis, and thyroid dysfunction, leading to adverse effects on host health (55–58). Importantly, liver, kidney, and thyroid function regulate skeletal metabolism (55, 59–61). Histopathology studies did not detect minocycline-induced toxicity effects in the kidney or liver of male SPF mice (Figure 3F). Serum clinical chemistry analyses ruled out minocycline-induced changes in mineral homeostasis (calcium, phosphorus) and markers for compromised kidney and liver function (blood urea nitrogen, albumin, alanine aminotransferase, alkaline phosphatase, total protein) (Figure 3G). Minocycline did not alter the serum levels of thyroid-stimulating hormone (TSH) in male SPF mice (Figure 3H). These findings support that minocycline did not cause cytotoxic effects in the kidney, liver, or thyroid of male SPF mice.

Liver weight per body weight was decreased in minocycline- versus vehicle-treated male SPF mice (Figure 3I). In line with prior reports showing that liver weight correlates with alterations in hepatic glycogen content (62, 63), the minocycline-induced decreased liver weight was associated with reduced hepatic glycogen (Figure 3, J and K). Notably, prior reports have shown that antibiotic disruption of the gut microbiota reduces liver weight per body weight in rodents (64, 65).

### Minocycline effects on skeletal maturation and liver are microbiota dependent.

**Figure 4:**
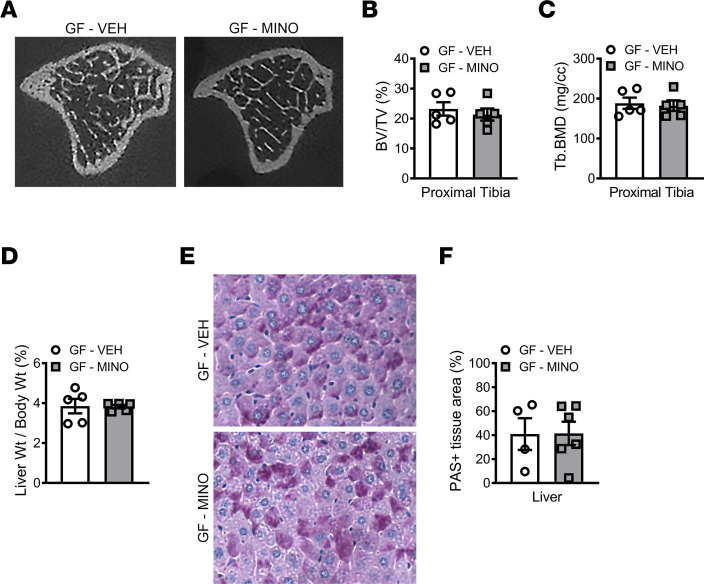
Minocycline effects on skeletal maturation and liver are microbiota dependent. Male C57BL/6T germ-free (GF) mice were administered vehicle control (VEH) or minocycline (MINO) from age 6 to 12 weeks; euthanized at age 12 weeks. Micro-CT analysis of proximal tibia trabecular bone; *n* = 5/group: (**A**) representative images; (**B**) bone volume per tissue volume (BV/TV); (**C**) trabecular bone mineral density (Tb.BMD). (**D**) Liver weight per body weight; *n* = 5/group. Periodic acid–Schiff–stained (PAS-stained) median liver lobe sections; *n* = 4–6/group: (**E**) representative images (original magnification, 200×), (**F**) PAS^+^ area per tissue area (%). Unpaired 2-tailed *t* test; reported as mean ± SEM.

To discern if minocycline treatment effects observed in male SPF mice are dependent on the microbiota, we administered minocycline to male C57BL/6T GF mice from age 6 to 12 weeks (Figure 1A). 16S analyses of colonic contents validated that GF mice were devoid of microbes (Supplemental Figure 3A). Minocycline did not alter the body weight, body length, or tibia length in male GF mice (Supplemental Figure 3, B–E). Micro-CT analysis in the tibia demonstrated that minocycline did not affect trabecular bone (Figure 4, A–C, and Supplemental Figure 3, F–H) or cortical bone (Supplemental Figure 3, I–L) outcomes in male GF mice. There were no differences in serum TSH levels in minocycline- versus vehicle-treated male GF mice (Supplemental Figure 3M). Liver weight per body weight (Figure 4D) and hepatic glycogen content (Figure 4, E and F) were similar in minocycline- versus vehicle-treated male GF mice. Study outcomes in the GF murine model support that minocycline effects on the skeleton and liver are dependent on the gut microbiota. Importantly, results reported here are consistent with prior antibiotic administration studies in GF animals; antibiotic effects on postnatal growth and maturation depend on the gut microbiota (66, 67).

We simulated primary osteoblastic cells in culture with minocycline (Supplemental Figure 4) to rule out direct minocycline effects on osteoblastogenesis. Bone marrow stromal cells (BMSCs) were isolated from untreated male 10-week-old C57BL/6T SPF mice and cultured in osteogenic media to differentiate the cells into osteoblasts. Osteoblast cultures were then supplemented with no treatment control or minocycline for 14 days. A 1.25 μg/mL minocycline dose was chosen because pharmacokinetics studies have shown that orally administering a therapeutic minocycline dose results in plasma minocycline concentrations of at least 1.0 μg/mL for 8 to 12 hours (68, 69). Minocycline treatment did not alter the mineralization potential of osteoblasts (Supplemental Figure 4), which is in agreement with prior reports showing that therapeutic plasma levels of minocycline do not suppress the osteogenic potential of cultured osteoblasts (39, 69).

### Minocycline has lasting detrimental effects on the skeleton and liver.

**Figure 5:**
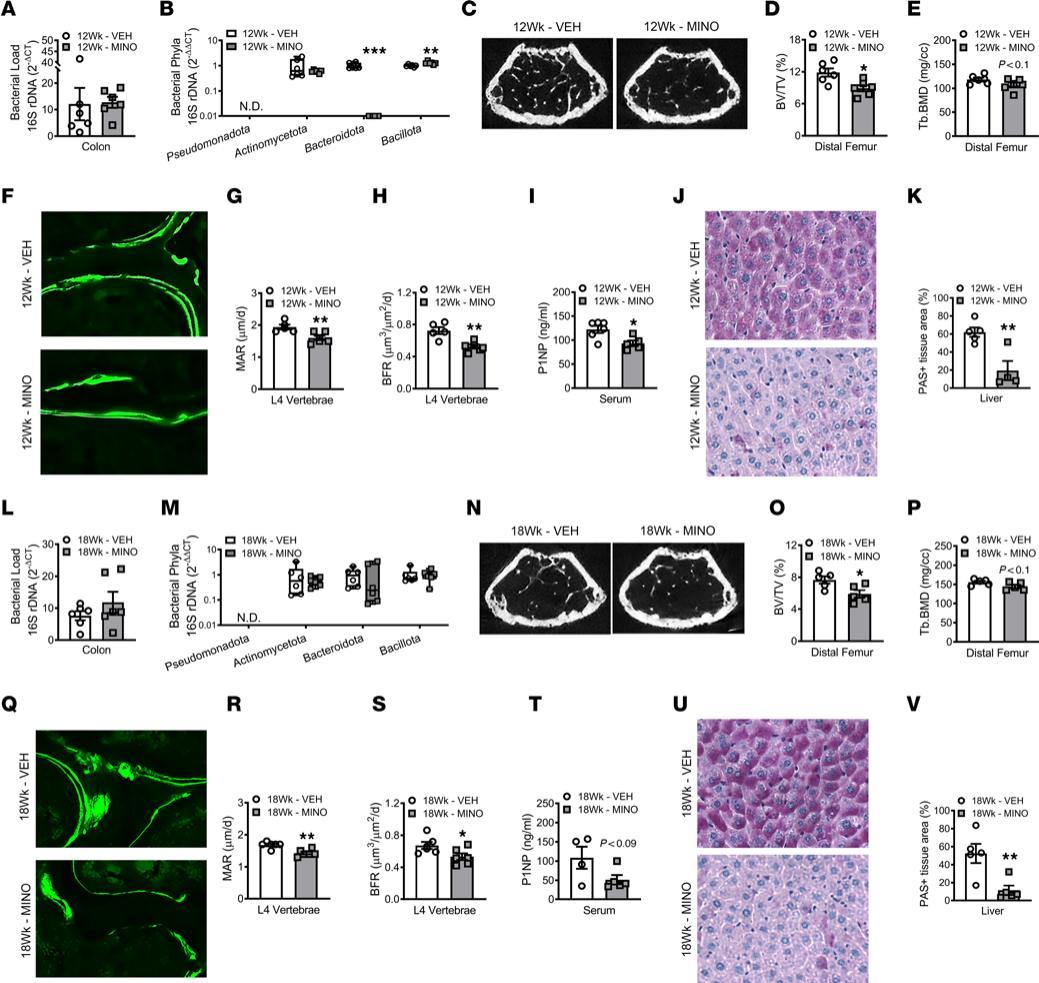
Minocycline has lasting detrimental effects on the skeleton and liver. Female C57BL/6T specific pathogen–free (SPF) mice were administered vehicle control (VEH) or minocycline (MINO) from age 6 to 12 weeks; euthanized at (**A**–**K**) age 12 weeks and (**L**–**V**) age 18 weeks. qRT-PCR 16s rDNA analysis of colonic contents evaluating bacterial load in (**A**) 12-week-old mice and (**L**) 18-week-old mice; *n* = 6/group. Bacterial load determined by normalizing the universal 16S gene to a bacterial DNA standard; quantification by the 2^-ΔCT^ method. qRT-PCR 16s rDNA analysis of colonic contents evaluating bacterial phyla in (**B**) 12-week-old mice and (**M**) 18-week-old mice; *n* = 6/group. Phylum outcomes determined by normalizing phyla genes to the universal 16S gene; quantification via the 2^-ΔΔCT^ method. Micro-CT analysis of distal femur trabecular bone in (**C**–**E**) 12-week-old mice (*n* = 6/group) and (**N**–**P**) 18-week-old mice (*n* = 5/group): (**C** and **N**) representative images; (**D** and **O**) bone volume per tissue volume (BV/TV); (**E** and **P**) trabecular bone mineral density (Tb.BMD). Dynamic histomorphometric analysis of trabecular bone formation indexes in L4 vertebra of (**F**–**H**) 12-week-old mice and (**Q**–**S**) 18-week-old mice; calcein administered 5 and 2 days prior to sacrifice; *n* = 5–6/group: (**F** and **Q**) representative images (original magnification, 200×); (**G** and **R**) mineral apposition rate (MAR); (**H** and **S**) bone formation rate (BFR). N-terminal propeptide of type 1 procollagen (P1NP) serum ELISA in (**I**) 12-week-old mice (*n* = 5–6/group) and (**T**) 18-week-old mice (*n* = 4–5/group). Periodic acid–Schiff–stained (PAS-stained) median liver lobe sections in (**J** and **K**) 12-week-old mice and (**U** and **V**) 18-week-old mice; *n* = 4–5/group: (**J** and **U**) representative images (original magnification, 200×), (**K** and **V**) PAS^+^ area per tissue area (%). Unpaired 2-tailed *t* test in 12-week-old mice and 18-week-old mice; reported as mean ± SEM; *P < 0.05 vs. VEH, **P < 0.01 vs. VEH, ***P < 0.001 vs. VEH.

To determine whether minocycline treatment effects on the microbiota, skeleton, and liver are sex dependent, female SPF mice were administered a clinically relevant dose of minocycline from age 6 to 12 weeks and euthanized at age 12 weeks (Figure 1B). Similar to study outcomes in male SPF mice (Figure 2), minocycline perturbed the gut microbiota and impaired pubertal/postpubertal skeletal maturation in female SPF mice at age 12 weeks (Figure 5, A–I). 16S rDNA analyses showed that the colonic contents in minocycline-treated mice had reduced Bacteroidota and increased Bacillota (formerly Firmicutes) (54) (Figure 5B). In line with findings observed in male SPF mice, somatic growth outcomes were similar in 12-week-old minocycline- versus vehicle-treated female SPF mice (Supplemental Figure 5, A–D). Similar to male SPF mice, trabecular bone volume fraction, bone mineral density (Figure 5, C–E), and trabecular number (Supplemental Figure 5E) were decreased in the distal femur of minocycline-treated female SPF mice at age 12 weeks. Cortical bone area fraction, cortical thickness, and cortical bone mineral density were similar in the femur mid-diaphysis of minocycline- versus vehicle-treated female SPF mice at age 12 weeks (Supplemental Figure 5, H–K). Biomechanical loading analysis revealed that minocycline therapy increased the stiffness (Supplemental Figure 5L) and reduced the ultimate deflection required to fracture the tibia (Supplemental Figure 5M). Considering both cortical and trabecular bone properties are an important determinant of bone strength (70–72), the impaired fracture resistance observed in 12-week-old minocycline-treated female SPF mice appears to be attributed to inferior trabecular bone properties. Minocycline therapy did not alter osteoclast (Supplemental Figure 5, N and O) or osteoblast (Supplemental Figure 5, P and Q) numbers lining trabecular bone in 12-week-old female SPF mice. Consistent with the suppressed osteogenic phenotype found in male SPF mice (Figure 2), minocycline blunted the trabecular mineral apposition and bone formation rates in lumbar vertebrae (Figure 5, F–H) and decreased serum P1NP levels (Figure 5I) in 12-week-old female SPF mice. Similar to male SPF mice, liver weight per body weight (Supplemental Figure 5S) and hepatic glycogen content (Figure 5, J and K) were decreased in minocycline- versus vehicle-treated female SPF mice at age 12 weeks.

To determine whether minocycline has sustained effects on the gut microbiota, skeleton, and liver, female SPF mice were administered minocycline versus vehicle treatment from age 6 to 12 weeks and euthanized at age 18 weeks (Figure 1B). Phylum-level shifts were not detected in the colonic bacteriome of minocycline- versus vehicle-treated female SPF mice at age 18 weeks (Figure 5M). The minocycline-induced impaired trabecular bone phenotype (Figure 5, N–P) and ultimate deflection required to fracture the tibia (Supplemental Figure 6M) persisted in female SPF mice at age 18 weeks. Cortical bone outcomes were not different in minocycline- versus vehicle-treated female SPF mice at age 18 weeks (Supplemental Figure 6, H–K). Six weeks after ceasing antibiotic administration, osteoclast numbers (Supplemental Figure 6, N and O) and osteoblast numbers (Supplemental Figure 6, P and Q) remained similar, and the blunted osteoblast function phenotype was sustained (Figure 5, Q–T) in minocycline-treated female SPF mice at age 18 weeks. The minocycline-induced decreased liver weight per body weight (Supplemental Figure 6S) and reduced hepatic glycogen content (Figure 5, U and V) also persisted 6 weeks after terminating antibiotic administration. These results reveal that minocycline therapy during the pubertal/postpubertal growth phase has lasting effects on the skeleton and the liver.

### Minocycline causes sustained shifts across taxa in the gut bacteriome.

**Figure 6:**
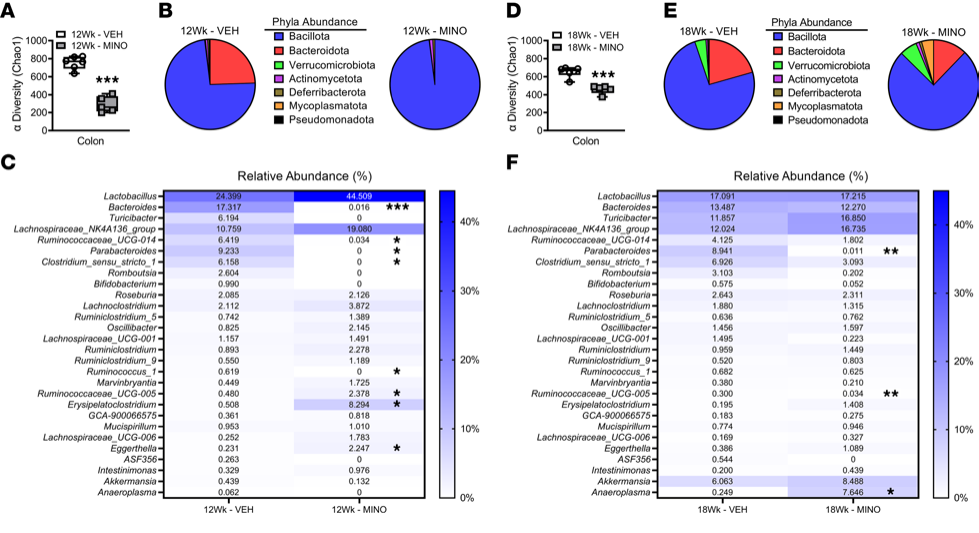
Minocycline causes sustained shifts across taxa in the colonic bacteriome. Female C57BL/6T specific pathogen–free (SPF) mice were administered vehicle control (VEH) or minocycline (MINO) from age 6 to 12 weeks; euthanized at (**A**–**C**) age 12 weeks and (**D**–**F**) age 18 weeks. Advanced 16S rDNA sequencing analysis of the colonic bacteriome; *n* = 5–6/group. Bacterial alpha diversity (Chao1 index) in (**A**) 12-week-old mice and (**D**) 18-week-old mice. Phyla relative abundance (%) in (**B**) 12-week-old mice and (**E**) 18-week-old mice. Genera relative abundance (%) in (**C**) 12-week-old mice and (**F**) 18-week-old mice. Unpaired 2-tailed *t* test with Holm-Šídák post hoc test in 12-week-old mice and 18-week-old mice; reported as mean ± SEM; *P < 0.05 vs. VEH, **P < 0.01 vs. VEH, ***P < 0.001 vs. VEH.

The resiliency of the gut microbiota to recover from antibiotic-induced dysbiosis is impaired by long-term antibiotic treatment regimens and antibiotic administration during critical phases of development (9–12). Considering sustained alterations in gut bacterial communities have been shown to have lasting detrimental effects on host health (4, 9–12), we performed advanced 16S rDNA sequencing of colonic contents from minocycline- versus vehicle-treated female SPF mice. Whereas our 16S qRT-PCR analysis was limited to evaluating targeted phylum-level changes (Figure 5, B and M), performing advanced 16S rDNA sequencing of colonic contents facilitated assessing comprehensive changes across bacterial taxa communities (Figure 6).

The alpha biodiversity, which measures the number of species present in an ecosystem, was reduced by 60% in the colonic bacteriome of minocycline- versus vehicle-treated female SPF mice at age 12 weeks (Figure 6A). Minocycline induced dysbiotic shifts across bacterial taxa in female SPF mice (Supplemental Data 1 — Bacterial Relative Abundance Table). In line with 16S qRT-PCR outcomes (Figure 5B), 16S rDNA-sequencing studies showed that minocycline treatment increased phylum Bacillota and decreased phylum Bacteroidota in the colonic bacteriome of female SPF mice at age 12 weeks (Figure 6B and Supplemental Figure 7A). Further, phylum Mycoplasamatota (formerly Tenericutes) (54) was undetectable in the colonic bacteriome of minocycline-treated 12-week-old female SPF mice (Figure 6B and Supplemental Figure 7A). Eight genera differed in the colonic contents of minocycline- versus vehicle-treated 12-week-old female SPF mice (Figure 6C). Minocycline treatment decreased the abundance of *Bacteroides*, *Ruminococcaceae UCG-014*, *Parabacteroides*, *Clostridium sensu stricto I*, and *Ruminococcus I*, and increased the abundance of *Ruminococcaceae UCG-005*, *Erysipelatoclostridium*, and *Eggerthella*, in female SPF mice at age 12 weeks (Figure 6C).

The minocycline-induced gut dysbiosis persisted 6 weeks after the cessation of antibiotic therapy. The alpha biodiversity of the colonic bacteriome remained reduced by 30% in minocycline- versus vehicle-treated female mice at age 18 weeks (Figure 6D). Corroborating 16S qRT-PCR outcomes (Figure 5M), 16S advanced sequencing showed that 6 weeks after terminating antibiotic therapy, the abundance of phyla Bacillota and Bacteroidota was similar in minocycline- versus vehicle-treated female SPF mice (Figure 6E and Supplemental Figure 7B). The abundance of phylum Mycoplasamatota was increased in minocycline- versus vehicle-treated female SPF mice at age 18 weeks (Figure 6E and Supplemental Figure 7B). At the genera level, the abundance of *Parabacteroides* was decreased and *Romboutsia* was increased (Figure 6F). Outcomes from advanced 16S rDNA sequencing studies show that long-term minocycline therapy during the pubertal/postpubertal growth phase impairs the colonic bacteriome’s ability to recover to a stable state.

### Minocycline causes persistent dysregulation of hepatic genes.

**Figure 7:**
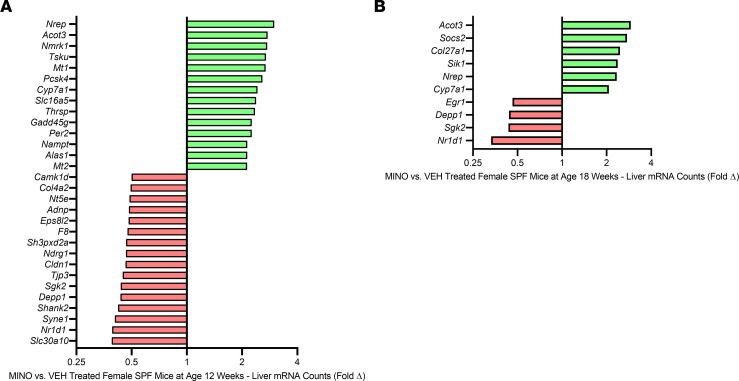
Minocycline causes persistent dysregulation of hepatic genes. Female C57BL/6T specific pathogen–free (SPF) mice were administered vehicle control (VEH) or minocycline (MINO) from age 6 to 12 weeks; euthanized at (**A**) age 12 weeks and (**B**) age 18 weeks. (**A**) Liver RNA-Seq analysis in MINO- vs. VEH-treated female SPF mice at age 12 weeks; *n* = 4/group. Unpaired 2-tailed *t* test with *P* < 0.05; data presented as fold difference vs. VEH. (**B**) Liver RNA-Seq analysis in MINO- vs. VEH-treated female SPF mice at age 18 weeks; *n* = 4/group. Unpaired 2-tailed *t* test with *P* < 0.05; data presented as fold difference vs. VEH.

Antibiotic perturbations of the healthy gut microbiota have been linked to changes in liver metabolism (13, 64, 65). Therefore, RNA-Seq was performed in left liver lobes of vehicle- versus minocycline-treated female SPF mice (Figure 7 and Supplemental Data 2 – Liver RNA-Seq Transcript Count Table). Thirteen genes were upregulated, and 17 genes were downregulated, by at least 2-fold, in the liver of minocycline- versus vehicle-treated female SPF mice at age 12 weeks (Figure 7A and Supplemental Table 1). Six weeks after ceasing antibiotic administration, 6 genes were increased and 4 genes were decreased by at least 2-fold in the liver of minocycline- versus vehicle-treated female SPF mice at age 18 weeks (Figure 7B and Supplemental Table 2). Minocycline treatment caused a sustained upregulation of 3 genes (*Cyp7a1*, *Nrep*, *Acot3*) and persistent downregulation of 3 genes (*Nr1d1*, *Sgk2*, *Depp1*) in female SPF mice at ages 12 and 18 weeks (Figure 7, A and B, and Supplemental Tables 1 and 2). CYP7A1 is the rate-limiting enzyme in the classic bile acid synthesis pathway, accounting for over 90% of hepatic primary bile acid synthesis (23, 24). Prior work has shown that antibiotic-induced disruptions of the indigenous gut bacteriome modify hepatic CYP7A1 expression and bile acid homeostasis (33, 34, 64). Therefore, subsequent studies were centered on discerning the role of bile acids in minocycline-induced gut dysbiosis effects on the skeleton.

### Minocycline causes a sustained disruption of the intestinal FXR/FGF15 axis.

CYP7A1-mediated bile acid synthesis is regulated by the intestinal FXR/FGF15 axis and local FXR signaling in the liver (23–29). Primary bile acids are synthesized in the liver and secreted into the intestine to facilitate digestion (23–29). Intestinal bacteria deconjugate bile acids and metabolize them into secondary bile acids (24–26, 31, 32). Microbial biotransformation of bile acids modifies their ability to function as agonists or antagonists at FXR (24–26, 31, 32). Bile acid activation of enterocyte/FXR induces the synthesis of intestinal FGF15, which signals in the liver to suppress CYP7A1-mediated bile acid synthesis (23–29). In addition to bile acid activation of the intestinal FXR/FGF15 axis, bile acid signaling at hepatocyte/FXR also regulates CYP7A1. Bile acid activation of hepatocyte/FXR induces small heterodimer partner (SHP), which functions as a local feedback mechanism that suppresses CYP7A1-mediated bile acid synthesis (23–29).

**Figure 8:**
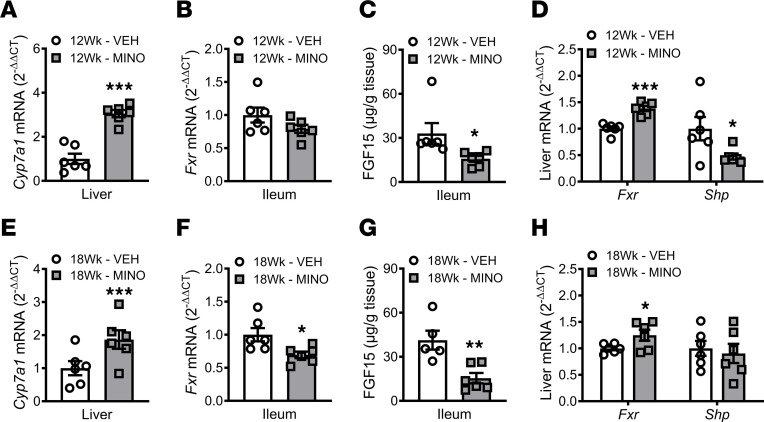
Minocycline causes a sustained disruption of the intestinal FXR/FGF15 axis. Female C57BL/6T specific pathogen–free (SPF) mice were administered vehicle-control (VEH) or minocycline (MINO) from age 6 to 12 weeks; euthanized at (**A**–**D**) age 12 weeks and (**E**–**H**) age 18 weeks. Liver qRT-PCR analysis of *Cyp7a1* in (**A**) 12-week-old mice and (**E**) 18-week-old mice; *n* = 6/group. Ileum qRT-PCR analysis of *Fxr* in (**B**) 12-week-old mice and (**F**) 18-week-old mice; *n* = 6/group. Ileum ELISA analysis of FGF15 in (**C**) 12-week-old mice and (**G**) 18-week-old mice; *n* = 5–6/group. Liver qRT-PCR analyses of *Fxr* and *Shp* in (**D**) 12-week-old mice and (**H**) 18-week-old mice; *n* = 6/group. qRT-PCR outcomes analyzed by the 2^-ΔΔCT^ method; normalized to *Gapdh*. Unpaired 2-tailed *t* test; reported as mean ± SEM; *P < 0.05 vs. VEH, **P < 0.01 vs. VEH, ***P < 0.001 vs. VEH.

qRT-PCR analysis in livers validated RNA-Seq outcomes (Figure 7, A and B), demonstrating hepatic *Cyp7a1* was upregulated by minocycline therapy in female SPF mice at ages 12 weeks (Figure 8A) and 18 weeks (Figure 8E). Minocycline treatment in female SPF mice did not alter ileal *Fxr* expression at age 12 weeks (Figure 8B) but led to a decrease in ileal *Fxr* expression at age 18 weeks (Figure 8F). Ileal FGF15 levels were decreased (Figure 8C), and hepatic *Shp* (*Nr0b2*) expression was downregulated (Figure 8D) in minocycline- versus vehicle-treated female SPF mice at age 12 weeks. These data imply that minocycline actions increasing liver *Cyp7a1* in 12-week-old female SPF mice are attributed to disruption of the intestinal FXR/FGF15 axis and suppression of hepatic FXR/SHP signaling. Six weeks after terminating antibiotics, the increased liver *Cyp7a1* expression (Figure 8E) and reduced ileal FGF15 levels (Figure 8G) persisted in minocycline-treated female SPF mice at age 18 weeks. However, hepatic *Shp* expression was not different in minocycline- versus vehicle-treated female SPF mice at age 18 weeks (Figure 8H). Therefore, the minocycline-induced persistent upregulation of liver *Cyp7a1* appears to be mediated by a sustained disruption of the intestinal FXR/FGF15 axis following the cessation of antibiotic therapy. Importantly, intestinal FGF15 supports hepatic glycogen accumulation and relative liver weight (27–29). Thus, the decreased liver weight (Supplemental Figure 5S and Supplemental Figure 6S) and reduced hepatic glycogen phenotype observed in minocycline-treated SPF mice (Figure 5, J, K, U, and V) indirectly supports that minocycline disrupts the intestinal FXR/FGF15 axis.

Since hepatic *Cyp7a1* was increased following minocycline therapy (Figure 7 and Figure 8, A and E), we evaluated the expression of other key liver bile acid synthesis enzymes (Supplemental Figure 8, A and C). Minocycline treatment did not alter downstream enzymes in the classical bile acid synthesis pathway (*Cyp8b1*, *Cyp27a1*), nor did it affect enzymes involved in the alternative bile acid synthesis pathway (*Cyp27a1*, *Cyp7b1*) (Supplemental Figure 8, A and C) (23–26). Primary bile acids are conjugated with glycine or taurine by the enzymes BACS and BAAT prior to being secreted from the liver (23–26). Minocycline therapy did not change the expression of *Bacs* (*Slc27a5*) or *Baat* in the livers of minocycline- versus vehicle-treated female SPF mice (Supplemental Figure 8, B and D). Outcomes in female SPF mice revealed that minocycline induced a stable suppression of ileal FGF15 and persistent upregulation of liver *Cyp7a1*, which suggest that minocycline provokes lasting dysbiotic shifts in the gut bacteriome that cause an enduring disruption of the intestinal FXR/FGF15 axis.

### Minocycline upregulates conjugated bile acids in systemic circulation (serum).

**Figure 9:**
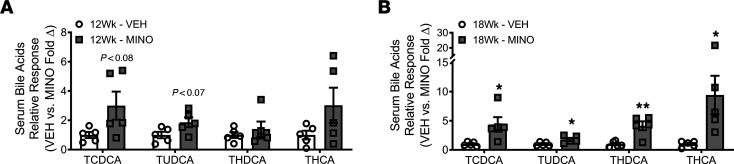
Minocycline upregulates conjugated bile acids in systemic circulation (serum). Female C57BL/6T specific pathogen–free (SPF) mice were administered vehicle control (VEH) or minocycline (MINO) from age 6 to 12 weeks; euthanized at (**A**) age 12 weeks and (**B**) age 18 weeks. Mass spectrometry serum bile acid relative response analysis in MINO- versus VEH-treated female SPF mice at (**A**) age 12 weeks and (**B**) age 18 weeks; *n* = 5/group. Presented as fold difference versus VEH; unpaired *t* test; reported as mean ± SEM; *P < 0.05 vs. VEH, **P < 0.01 vs. VEH. TCDCA, taurochenodeoxycholic acid; TUDCA, tauroursodeoxycholic acid; THDCA, taurohyodeoxycholic acid; THCA, taurohyocholic acid.

Disruption of the intestinal FXR/FG15 axis upregulates hepatic bile acid synthesis and dysregulates systemic bile acid homeostasis (27–29, 73–75). Therefore, we evaluated minocycline-induced changes in serum bile acids using liquid chromatography-tandem mass spectrometry separation and electrospray negative ionization–triple quadrupole multiple reaction monitoring methods. The approach allowed us to assess 20+ bile acids in serum (Supplemental Data 3 – Serum Bile Acid Proteomic Analysis and Figure 9). Minocycline increased TCDCA, TUDCA, THDCA, and THCA in the serum of female SPF mice. This collection of bile acids was trending upward at age 12 weeks (Figure 9A) and significantly increased at age 18 weeks (Figure 9B) in minocycline- versus vehicle-treated female SPF mice. The altered serum bile acid pool detected in minocycline-treated SPF mice comprised taurine-conjugated bile acids that primarily act as antagonists at FXR (Table 1) (35, 36, 76–78).

Bile acids act as effector molecules within the enterohepatic loop and enter the serum to signal at distant sites (2, 3, 79). Osteoblasts express FXR (80, 81); however, it is unknown whether serum bile acid signaling at osteoblast/FXR regulates bone metabolism. Osteoblastic cells derived from global *Fxr*-knockout (*Nr1h4*-knockout) mice have reduced osteogenic potential (81), which implies that FXR positively regulates osteogenesis. Stimulating osteoblastic cells with chenodeoxycholic acid (CDCA), a primary bile acid and potent endogenous FXR agonist, promotes osteogenesis (80–82). Studies from the gut and liver fields have shown that different bile acids function as potent agonists, weak/partial agonists, or antagonists at FXR and that the consortium of bile acids present in a local environment regulates FXR signaling (24, 25, 76–78). Fujimori et al. subjected osteoblastic cells to stimulation with CDCA alone versus costimulation with a known FXR antagonist (82). The FXR antagonist neutralized CDCA’s pro-osteogenic actions, which importantly discerns that the consortium of bile acids, not the presence of a specific bile acid, regulates FXR signaling processes in osteoblastic cells (82).

### Minocycline-induced alterations in serum bile acids suppress osteogenesis through attenuating osteoblast/FXR signaling.

**Figure 10:**
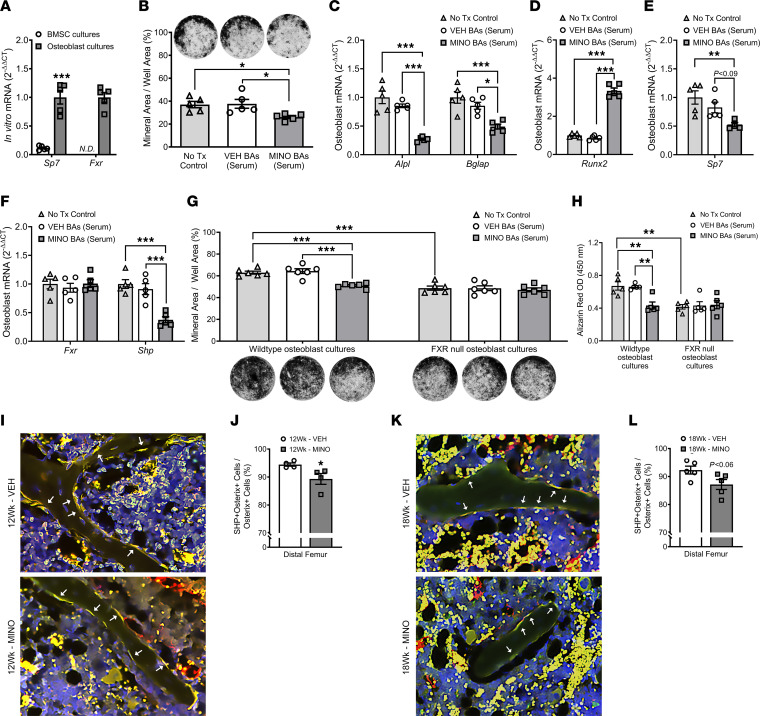
Minocycline-induced alterations in serum bile acids suppress osteogenesis through attenuating osteoblast/FXR signaling. (**A**–**E**) Bone marrow stromal cells (BMSCs) were isolated from untreated 10-week-old female C57BL/6T specific pathogen–free (SPF) mice. (**A**) BMSCs were cultured in base media (α-MEM, 10% FBS, 1% PSG) versus osteogenic media (base media, 50 mg/mL ascorbic acid, 10 mM β-glycerophosphate) to evaluate differences in pre-osteoblastic cells versus mature osteoblastic cells. qRT-PCR: *Sp7* and *Fxr*; *n* = 5/group. Unpaired 2-tailed *t* test; reported as mean ± SEM; ***P < 0.001. (**B**–**F**) Mature osteoblasts were stimulated with no treatment control (No Tx Control) or the altered serum bile acid profiles from minocycline-treated (MINO BAs) versus vehicle-treated (VEH BAs) female SPF mice; *n* = 5/group. (**B**) von Kossa assay; representative images and mineral area per well area (%) analysis. qRT-PCR: (**C**) *Akp2/Alpl*, *Bglap/Ocn*, (**D**) *Runx2*, (**E**) *Sp7*, (**F**) *Fxr*, *Shp*. One-way ANOVA with Tukey’s post hoc test; reported as mean ± SEM; *P < 0.05, **P < 0.01, ***P < 0.001. (**G** and **H**) BMSCs were isolated from untreated 10-week-old female C57BL/6J FXR-knockout and wild-type mice. Mature osteoblasts were stimulated with no Tx control, MINO serum BAs, or VEH serum BAs; *n* = 5/group. (**G**) von Kossa assay; representative images and mineral area per well area (%) analysis. (**H**) Alizarin red assay, optical density (OD) 450 nm. Two-way ANOVA with Tukey’s post hoc test; reported as mean ± SEM; **P < 0.01, ***P < 0.001. (**I**–**L**) Female C57BL/6T SPF mice were administered vehicle control (VEH) or minocycline (MINO) from age 6 to 12 weeks; euthanized at (**I** and **J**) age 12 weeks and (**K** and **L**) age 18 weeks. Immunofluorescence analysis of dual-labeled SHP^+^osterix^+^ cuboidal osteoblasts lining trabecular bone in the distal femur (green, SHP-FITC; red, osterix–rhodamine; blue, DAPI); *n* = 4/group: (**I** and **K**) representative images (original magnification, 200×), arrows indicate SHP^+^osterix^+^ osteoblasts; (**J** and **L**) SHP^+^osterix^+^ cells per osterix^+^ cells (%). Unpaired 2-tailed *t* test; reported as mean ± SEM; *P < 0.05 vs. VEH.

We isolated BMSCs from untreated 10-week-old female C57BL/6T wild-type SPF mice in order to elucidate differences in *Fxr* expression across osteoblast lineage cells (Figure 9A). BMSCs were cultured in base media (α-MEM, 10% FBS, 1% penicillin-streptomycin-glutamine [PSG]) versus osteogenic media (α-MEM, 10% FBS, 1% PSG, 50 mg/mL ascorbic acid, 10 mM β-glycerophosphate) to evaluate alterations in *Fxr* expression in BMSC osteoprogenitor cells versus committed osteoblastic cells; *Sp7* (osterix) was used as a marker for commitment to the osteoblast lineage (Figure 10A). *Fxr* was highly expressed in osteoblasts but undetectable in BMSC osteoprogenitor cells (Figure 10A). These data support the notion that serum bile acids affect osteoblastogenesis in vivo through FXR signaling at committed osteoblast lineage cells.

We performed in vitro osteoblastogenesis studies to elucidate the relationship between the minocycline-induced suppressed osteoblast phenotype (Figure 5) and altered serum bile acid profile (Figure 9) found in female SPF mice. BMSCs from untreated 10-week-old female C57BL/6T wild-type SPF mice were cultured under osteogenic conditions to differentiate the cells into mature osteoblasts. We then stimulated the mature osteoblast cells with no treatment control (no Tx control) or the altered serum bile acid profiles detected in minocycline-treated female SPF mice (MINO serum BAs) versus vehicle-treated female SPF mice (VEH serum BAs). von Kossa mineralization assays (Figure 10B) and qRT-PCR studies (Figure 10, C–E) were carried out to assess changes in osteoblast differentiation and function. Comparison of osteoblast cultures simulated with no Tx control or VEH serum BAs to MINO serum BAs revealed that the altered serum bile acid profile from minocycline-treated SPF mice suppressed osteoblast function. Mineralization potential was blunted by greater than 30% (Figure 10B), and gene markers for osteoblast function (*Akp2/Alpl*, *Bglap/Ocn*) were decreased by more than 1-fold (Figure 10C) in cultures stimulated with MINO serum BAs. Stimulation with minocycline serum bile acids substantially upregulated *Runx2* (Figure 10D) and suppressed *Sp7* (Figure 10E). Considering RUNX2 is strongly expressed in immature osteoblasts and decreases during osteoblast maturation, which is induced by SP7 (83), these transcription factor findings support that the minocycline serum bile acid profile suppressed osteoblast maturation. Study outcomes showing that the altered serum bile acid profile from minocycline-treated female SPF mice (Figure 9) suppressed osteogenesis in vitro (Figure 10, B–E) recapitulated the suppressed osteoblast phenotype found in vivo (Figure 5).

The altered serum bile acid pool detected in minocycline-treated female SPF mice predominantly comprised bile acids that act as FXR antagonists (Figure 9) (35, 36, 76–78), which supports the premise that minocycline-induced changes in serum bile acids suppress osteogenesis through attenuating FXR signaling in osteoblast cells. FXR activation modulates cellular processes in diverse hepatic and extrahepatic cells through the induction of SHP (84, 85). SHP, an atypical orphan nuclear receptor, is a positive regulator of osteoblast differentiation and function (86). Therefore, we evaluated *Fxr* and *Shp* gene expression in the mRNA isolated from the mature osteoblastic cells stimulated for 10 days with no Tx control, MINO serum BAs, or VEH serum BAs (Figure 10F). *Shp* was profoundly downregulated in osteoblast cultures stimulated with MINO serum BAs (Figure 10F), which is consistent with the observed in vitro findings that the altered serum bile acid profile from minocycline-treated SPF mice suppressed the osteogenic potential of mature osteoblast cells (Figure 10, B–E). Considering the altered serum bile acid pool detected in minocycline-treated SPF mice predominantly comprised bile acids that act as FXR antagonists (Figure 9) (35, 36, 76–78), and that FXR activation (80–82) and downstream SHP signaling (86) in osteoblasts promote osteogenesis, in vitro study outcomes (Figure 10, B–F) imply that minocycline-induced alterations in serum bile acids suppress osteogenesis by attenuating osteoblast/FXR signaling.

To delineate the role of osteoblast/FXR signaling in minocycline-induced alterations in serum bile acids’ suppression of osteogenesis, BMSCs were isolated from untreated 10-week-old female C57BL/6 FXR-knockout and wild-type SPF mice (Figure 10, G and H, and Supplemental Figure 9A). BMSCs were cultured under osteogenic conditions to differentiate the cells into osteoblasts, then stimulated for 14 days with no treatment control (no Tx control) or the altered serum bile acid profiles detected in minocycline-treated SPF mice (MINO serum BAs) versus vehicle-treated SPF mice (VEH serum BAs). von Kossa and alizarin red mineralization assays were carried out to evaluate differences in osteoblast function. Corroborating prior reports that *Fxr*-knockout mouse–derived osteoblasts have reduced osteogenic potential (81), mineralization was decreased in no Tx control–stimulated FXR-null osteoblasts versus wild-type osteoblasts (Figure 10, G and H, and Supplemental Figure 9A). Stimulation with MINO serum BAs suppressed the mineralization potential in wild-type osteoblasts but not FXR-null osteoblasts (Figure 10, G and H, and Supplemental Figure 9A).

Based on findings that *Shp* expression was decreased in osteoblast cultures treated with the serum bile acid profile found in minocycline-treated SPF mice, in situ immunofluorescence staining and histomorphometric analysis were performed to evaluate SHP expression in osterix^+^ osteoblasts lining trabecular bone in vehicle- versus minocycline-treated mice. Minocycline therapy reduced the frequency of SHP^+^ osteoblasts in female SPF mice at age 12 weeks (Figure 10, I and J) and age 18 weeks (Figure 10, K and L). Similar to female SPF mice, minocycline reduced the frequency of SHP^+^ osteoblasts in male SPF mice (Supplemental Figure 9, B and C). Minocycline treatment did not alter the frequency of SHP^+^ osteoblasts in male GF mice (Supplemental Figure 9, D and E), which supports that minocycline suppression of osteoblast SHP expression is dependent on the microbiota.

## Discussion

This report demonstrates that minocycline-induced gut dysbiosis during the pubertal/postpubertal growth phase has lasting detrimental effects on osteogenesis and skeletal maturation. Our prior work revealed that gut microbiota effects on bone are mediated through a signaling axis that involves communication with the liver, which supported a novel gut/liver/bone axis (7, 22). Minocycline therapy caused a stable gut dysbiosis, persistent disruption of the FXR/FGF15 gut/liver endocrine axis, and sustained dysregulation of serum bile acids that suppressed osteogenesis through attenuated osteoblast/FXR signaling. This work builds upon the premise that gut microbiota effects on the skeleton are mediated in part through a gut/liver signaling axis and introduces bile acids as a potentially novel regulator of gut microbiota effects on bone metabolism.

Factors influencing the gut microbiota composition during critical phases of postnatal development have implications for health and disease throughout life (1, 4, 11, 12, 87). Early research suggested that the gut microbiota establishes an adult-like profile during the first 3 years of life (88, 89). More recent investigations have shown that the gut microbiota continues to develop toward an adult-like profile during puberty (90, 91), implying that perturbations in the gut microbiota during adolescence may have lifelong implications for host health. The gut microbiota’s ability to recover to a stable state following antibiotic-induced dysbiosis is impaired by antibiotic administration during critical developmental windows (9–12). Therefore, antibiotic-induced gut dysbiosis during the pubertal/postpubertal growth phase, a critical period for attaining peak skeletal bone mass (15–17), may have lasting effects on skeletal health and fracture risk.

Antibiotic-induced changes in the gut microbiota influence bone mass and skeletal biomechanical properties (13, 14, 92, 93). We have previously demonstrated that administering a non–clinically relevant antibiotic cocktail (vancomycin, imipenem/cilastatin, and neomycin) from age 6 to 12 weeks in C57BL/6T mice induces a gut dysbiosis that impairs pubertal/postpubertal skeletal maturation (14). This broad-spectrum antibiotic cocktail caused a hyperimmune response in the MLNs and spleen that upregulated circulating (serum) proinflammatory mediators, which enhanced osteoclastogenesis to impair bone mass accrual (14). The current study shows that administering a clinically relevant minocycline dose from age 6 to 12 weeks in C57BL/6T mice caused a gut dysbiosis that impaired skeletal maturation. However, this was not due to an upregulated proinflammatory response in gastrointestinal and systemic lymphoid tissues or enhanced osteoclastogenesis. Instead, minocycline disrupted the gut/liver bile acid axis and dysregulated serum bile acids, which suppressed osteoblast function to impair trabecular bone morphology and bone mass accrual.

The distal femur trabecular bone volume fraction decreased by roughly 35% in vehicle-treated female C57BL/6T SPF mice from age 12 to 18 weeks, which is consistent with normal aging effects reported in the C57BL/6 skeleton (52). Similarly, the distal femur trabecular bone volume fraction decreased by about 34% from age 12 to 18 weeks in minocycline-treated female C57BL/6T SPF mice. These findings demonstrate that minocycline treatment during pubertal/postpubertal growth impairs trabecular bone maturation, and the skeleton is unable to recover following antibiotic therapy.

Disruption of the intestinal FXR/FGF15 axis occurs when there is attenuated bile acid activation of enterocyte/FXR, which suppresses intestinal FGF15 synthesis (25–29). Diminished FGF15 signaling at hepatocyte/FGFR4 upregulates CYP7A1-mediated liver bile acid synthesis and dysregulates systemic bile acid homeostasis (25–29). Disruption of the intestinal FXR/FGF15 axis has been shown to dysregulate systemic bile acid homeostasis, including increased circulating (serum) bile acids (27–29, 73–75). Consistent with these prior reports, minocycline disruption of the intestinal FXR/FGF15 axis increased serum conjugated bile acids. Under physiological states, the serum bile acid profile typically reflects the intestinal bile acid pool since these bile acids escape hepatic recovery from enterohepatic circulation (23, 94, 95). Therefore, we speculate that minocycline-induced increases in bile acids that act as FXR antagonists inhibited the intestinal FXR/FGF15 axis.

Bacteria encode unique bile salt hydrolases (BSHs) that differentially deconjugate bile acids, which explains why changes in gut bacterial composition alter the conjugation status of the intestinal bile acid pool (25, 26, 31, 32). Notably, BSH deconjugation of intestinal bile acids supports the activation of the intestinal FXR/FGF15 axis (35, 36, 96). Considering that minocycline treatment did not alter hepatic bile acid conjugation enzymes (BACS, BAAT), the increased conjugated bile acids observed in minocycline-treated SPF mice appear to be attributed to shifts in the colonic bacteriome that suppress BSH activity.

Prior murine antibiotic treatment studies support the concept that antibiotics disrupt the intestinal FXR/FGF15 axis through modulating intestinal BSH activity (33, 34). These investigations demonstrated that a 3-day course of ampicillin (33) or antibiotic cocktail (bacitracin, neomycin, and streptomycin) (34) increased intestinal conjugated bile acids that are FXR antagonists, which suppressed intestinal *Fgf15* and upregulated hepatic *Cyp7a1*. Recognizing that BSHs deconjugate intestinal bile acids to support the activation of the intestinal FXR/FGF15 axis (35, 36, 96), findings from a high-throughput screen identifying tetracyclines as potent BSH inhibitors (>90% inhibition) (97) supports the notion that tetracycline antibiotics disrupt the intestinal FXR/FGF15 axis through depleting/inhibiting bacterial BSHs.

Systemic minocycline therapy has been shown to cause shifts in the healthy gut microbiome of humans and rodents (44–47). Systemic minocycline administration to healthy young adults decreases the biodiversity and induces lasting genera-level shifts in the gut bacteriome (44). Administering a clinically relevant minocycline dose to C57BL/6T SPF mice from age 6 to 12 weeks caused sex-dependent phylum-level shifts in the gut bacteriome. Whereas minocycline downregulated the phyla Actinomycetota and Bacteroidota in male 12-week-old SPF mice, minocycline suppressed phylum Bacteroidota and upregulated phylum Bacillota in female SPF mice at age 12 weeks. Minocycline therapy similarly suppressed osteoblast function to impair bone mass accrual and skeletal maturation in male and female SPF mice. We speculate that sex-steroid hormone differences contribute to minocycline-induced sex-dependent effects on the gut bacteriome. This highlights the need for future metagenomic studies designed to evaluate minocycline sex-dependent effects on the gut bacteriome and skeleton.

Antibiotic-induced decreased biodiversity and compositional changes in the gut microbiome are associated with disruptions in host health (4, 9–12). Advanced 16S rDNA sequencing of the colonic bacteriome of female SPF mice at age 12 and 18 weeks revealed that minocycline therapy caused a sustained reduction of the alpha biodiversity and persistent depletion of genus *Parabacteroides*. Minocycline therapy depleted the abundance of genus *Parabacteroides* from roughly 9% to 0% in the colonic bacteriome of female SPF mice at age 12 and 18 weeks. The profound depletion of the genus *Parabacteroides* suggests that species belonging to this genus contribute to minocycline effects on host health.

*Parabacteroides distasonis* has been characterized as a core member of both the human and murine gut microbiota (98, 99). Interestingly, individuals afflicted with gut/liver disorders have a decreased abundance of *P*. *distasonis* (100, 101), dysregulated bile acids, and increased prevalence of osteoporosis (102, 103). Administering *P*. *distasonis* to mice increases bile acid deconjugation and metabolism of primary bile acids into secondary bile acids, which leads to a bile acid profile that predominantly acts as FXR agonists (104). Further, mice receiving *P*. *distasonis* have elevated ileal FGF15 and reduced hepatic CYP7A1 expression (104), which implies *P*. *distasonis* supports activation of the intestinal FXR/FGF15 axis. This highlights that depletion of *P*. *distasonis* may contribute to minocycline’s dysregulation of the gut/liver bile acid axis and detrimental effects on the skeleton.

This report introduces the intestinal FXR/FGF15 axis and circulating (serum) bile acids as potentially novel regulators of gut microbiota effects on bone metabolism. Our study findings support that minocycline-induced alterations in serum bile acids suppress osteogenesis through attenuating osteoblast/FXR signaling. The authors recognize that bile acids can signal at several receptors expressed by osteoblasts, including FXR, G protein–coupled bile acid receptor 1, and vitamin D receptor (80, 81, 105, 106). Since the altered serum bile acid profile from minocycline-treated SPF mice did not affect the osteogenic potential of FXR-null osteoblasts, this implies that minocycline-induced alterations in serum bile acids suppress osteogenesis through FXR-dependent signaling. The authors acknowledge that in addition to bile acids, other gut microbiota metabolites may contribute to minocycline’s actions impairing skeletal maturation. Further research is necessary to elucidate whether other gut microbiota metabolites, such as short-chain fatty acids and/or tryptophan derivatives, play a role in minocycline’s effects on skeletal metabolism.

This research demonstrates that minocycline-induced dysbiosis of the gut bacteriome during the pubertal/postpubertal growth phase suppresses osteogenesis and impairs bone mass accrual in the maturing skeleton. Studies herein were designed to model the administration of minocycline as a systemic treatment for acne in adolescents. Appreciating that minocycline is also administered for extended durations as a systemic therapy for rosacea in adults (39, 42, 43), minocycline may have detrimental effects on bone remodeling and the maintenance of bone mass in the mature adult skeleton. In addition to minocycline, other tetracycline derivatives (i.e., doxycycline, sarecycline) are commonly prescribed as long-term, systemic therapies for dermatological conditions (38, 39, 42, 43). However, doxycycline and sarecycline effects on skeletal maturation and homeostasis are unclear. Findings reported herein highlight the need for clinical research evaluating the impact of long-term systemic tetracycline therapies on the gut/liver bile acid axis and skeletal metabolism.

## Methods

Detailed Supplemental Methods are available online. 16S advanced sequencing reads are available from the Sequence Read Archive database under the BioProject accession number PRJNA892153. RNA-Seq reads are available from the NCBI’s Gene Expression Omnibus database under the accession number GSE217632.

### Statistics.

Unpaired 2-tailed *t* tests were performed comparing outcomes in minocycline- versus vehicle-treated 12-week-old male SPF mice, minocycline- versus vehicle-treated 12-week-old male GF mice, minocycline- versus vehicle-treated 12-week-old female SPF mice, and minocycline- versus vehicle-treated 18-week-old female SPF mice. For advanced 16S rDNA-sequencing analysis, unpaired 2-tailed *t* tests with Holm-Šídák post hoc test were performed in minocycline- versus vehicle-treated 12-week-old female SPF mice and minocycline- versus vehicle-treated 18-week-old female SPF mice. One-way ANOVA with Tukey’s post hoc test was performed to compare wild-type osteoblasts stimulated with no Tx control, VEH serum BAs, and MINO serum BAs. Two-way ANOVA with Tukey’s post hoc test was performed to compare FXR-knockout osteoblasts and wild-type osteoblasts stimulated with no Tx control, VEH serum BAs, and MINO serum BAs. Analyses were carried out utilizing GraphPad Prism 9.3 (GraphPad Software). Data are reported as ± SEM. Significance was determined at *P* < 0.05. Power analysis was carried out in consultation with the Medical University of South Carolina (MUSC) Bioinformatics Core.

### Study approval.

Research was approved by the MUSC Institutional Animal Care and Use Committee and carried out in accordance with the NIH *Guide for Care and Use of Laboratory Animals* (National Academies Press, 2011) and with the Animal Research: Reporting of In Vivo Experiments guidelines.

## Author contributions

CMN conceived the study. MDC, AJW, JDHS, CW, and CMN contributed to study design. MDC, AJW, JDHS, VLG, JK, JEG, YW, HY, JIA, and CMN performed data acquisition and analysis. MDC, AJW, JDHS, WDH, JJL, AVA, and CMN interpreted the data. MDC and CMN drafted the manuscript. All authors approved the final version of the manuscript. MDC and CMN take responsibility for the integrity of the data analysis.

## Supplementary Material

Supplemental data

Supplemental data set 1

Supplemental data set 2

Supplemental data set 3

## Figures and Tables

**Table 1:**
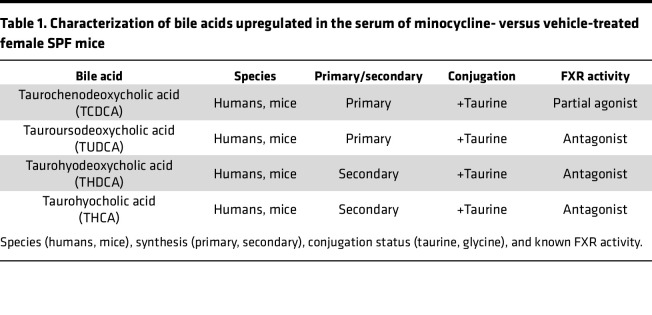
Characterization of bile acids upregulated in the serum of minocycline- versus vehicle-treated female SPF mice

## Version 1 11/22/2022

In-Press Preview

## Version 2 01/10/2023

Electronic publication
