# Supplementary material for: Minocycline-induced disruption of the intestinal FXR/FGF15 axis impairs osteogenesis in mice
Source: JCI Insight. 2023 Jan 10;8(1):e160578. doi: 10.1172/jci.insight.160578 (PMC9870091; doi:10.1172/jci.insight.160578)
Supplement: Supplemental data [file jciinsight-8-160578-s081.pdf]

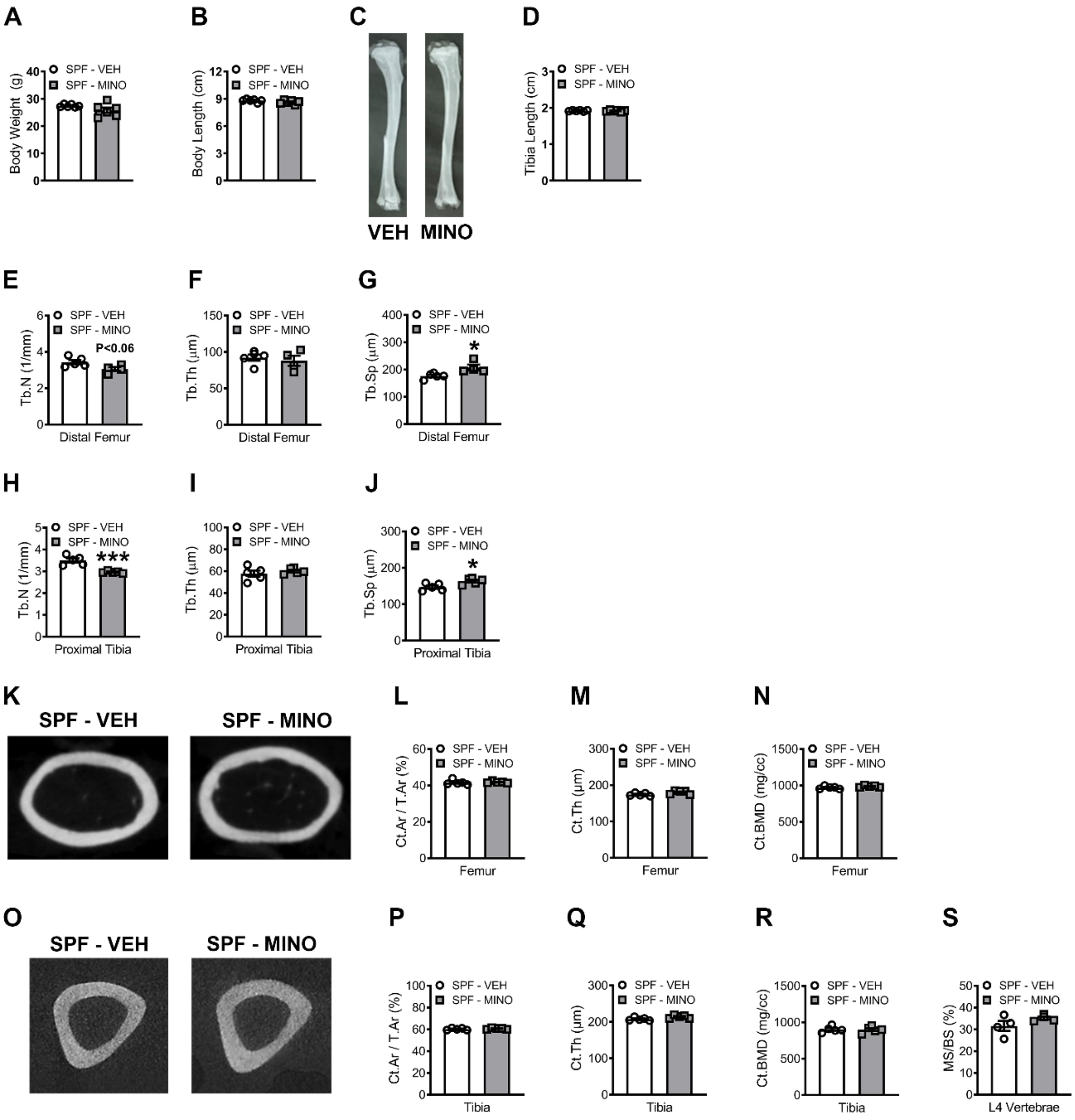

Supplemental Figure 1. Male C57BL/6T specific-pathogen-free (SPF) mice were administered vehicle-control (VEH) or minocycline (MINO) from age 6 to 12 weeks; euthanized at age 12 weeks. (**A**) Body weight; n=6/group. (**B**) Body length; n=6/group. (**C**) Representative faxitron images of tibia. (**D**) Tibia length; n=6/group. Micro-CT analysis of distal femur trabecular bone; n=4-5/group: (**E**) trabecular number (Tb.N); (**F**) trabecular thickness (Tb.Th); (**G**) trabecular separation (Tb.Sp). Micro-CT analysis of proximal tibia trabecular bone; n=5/group: (**H**) Tb.N; (**I**) Tb.Th; (**J**) Tb.Sp. Micro-CT analysis of femur mid-diaphysis cortical bone; n=4-5/group: (**K**) representative images; (**L**) cortical area per tissue area (Ct.Ar/T.Ar); (**M**) cortical thickness (Ct.Th); (N) cortical bone mineral density (Ct.BMD). Micro-CT analysis of tibia mid-diaphysis cortical bone; n=5/group: (**O**) representative images; (**P**) Ct.Ar/T.Ar; (**Q**) Ct.Th; (**R**) Ct.BMD. Dynamic histomorphometric analysis of trabecular bone formation indices in L4 vertebra; calcein administered 5 and 2 days prior to sacrifice; n=4/group: (**S**) mineralized surface per bone surface (MS/BS). Unpaired *t*-test; reported as mean ± SEM; *p<0.05 vs. VEH, ***p<0.001 vs. VEH.

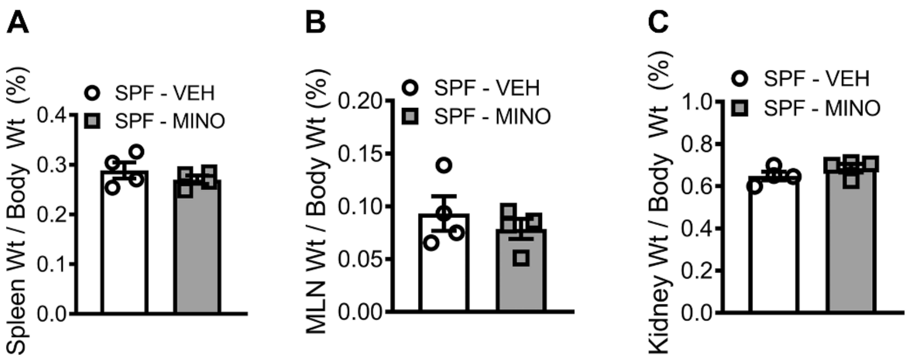

Supplemental Figure 2. Male C57BL/6T specific-pathogen-free (SPF) mice were administered vehicle-control (VEH) or minocycline (MINO) from age 6 to 12 weeks; euthanized at age 12 weeks. (**A**) Spleen weight per body weight; n=4/group. (**B**) Mesenteric lymph node (MLN) weight per body weight; n=4/group. (**C**) Kidney weight per body weight; n=4/group. Unpaired *t*-test; reported as mean ± SEM.

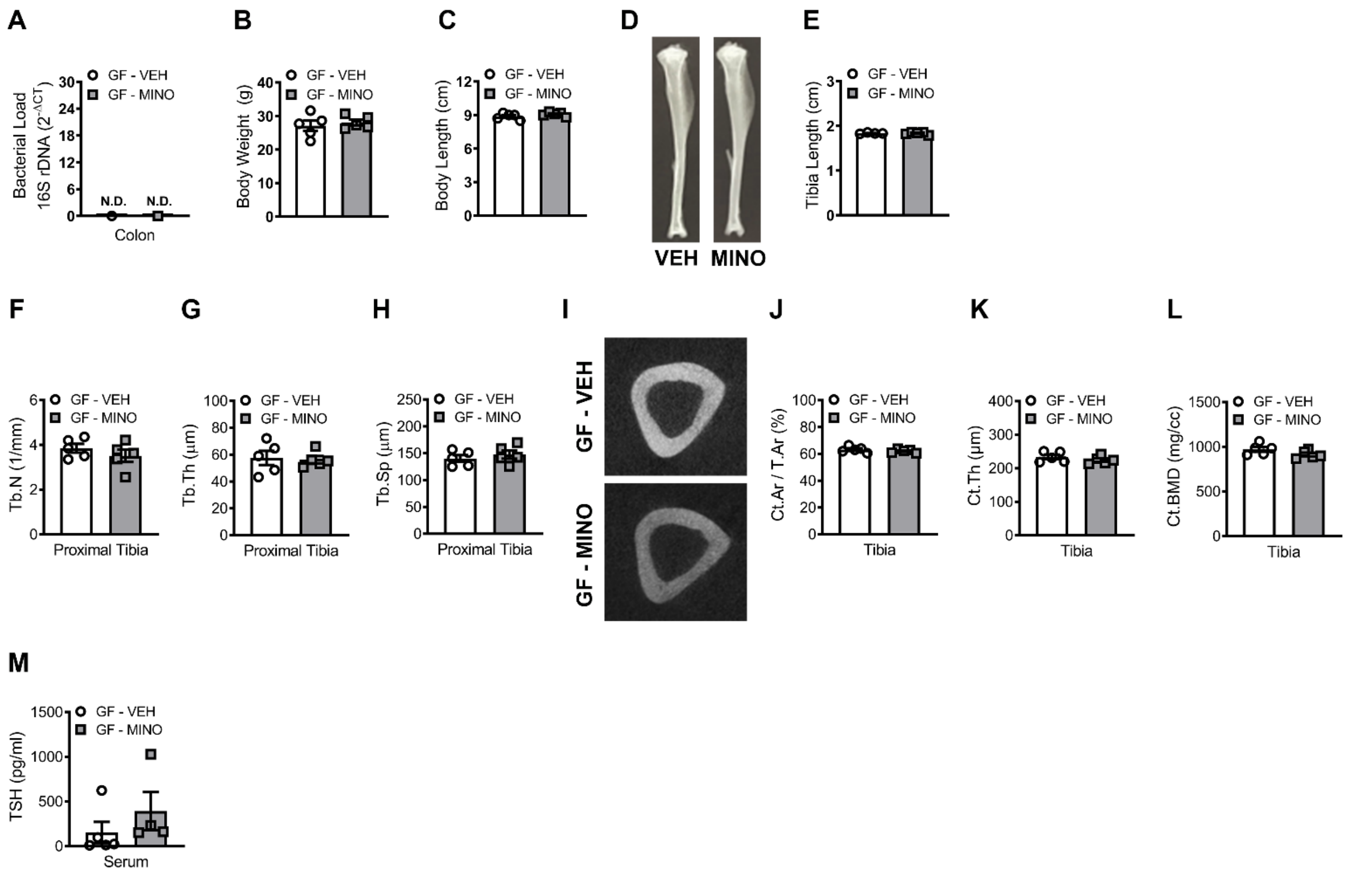

Supplemental Figure 3. Male C57BL/6T germ-free (GF) mice were administered vehicle-control (VEH) or minocycline (MINO) from age 6 to 12 weeks; euthanized at age 12 weeks. qRT-PCR 16s rDNA analysis of colonic contents evaluating (**A**) bacterial load; n=5/group. Bacterial load determined by normalizing the Universal 16S gene to a bacterial DNA standard; quantification by the $2^{-\Delta CT}$ method. (**B**) Body weight; n=5/group. (**C**) Body length; n=5/group. (**D**) Representative faxitron images of tibia. (**E**) Tibia length; n=5/group. Micro-CT analysis of proximal tibia trabecular bone; n=5/group: (**F**) trabecular number (Tb.N); (**G**) trabecular thickness (Tb.Th); (**H**) trabecular separation (Tb.Sp). Micro-CT analysis of tibia mid-diaphysis cortical bone; n=5/group: (**I**) representative images; (**J**) cortical area per tissue area (Ct.Ar/T.Ar); (**K**) cortical thickness (Ct.Th); (**L**) cortical bone mineral density (Ct.BMD). (**M**) Thyroid-stimulating hormone (TSH) serum ELISA; n=4-5/group. Unpaired *t*-test; reported as mean ± SEM.

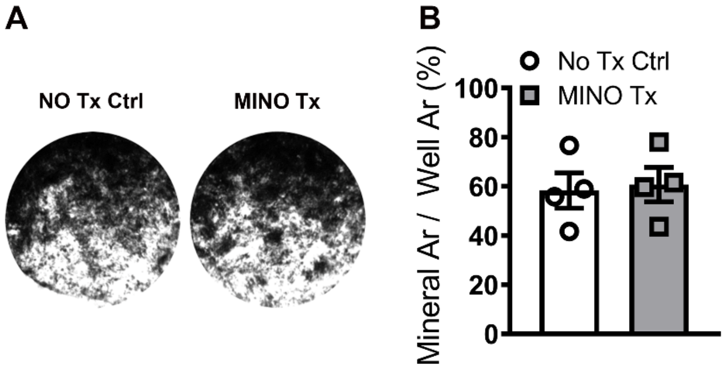

Supplemental Figure 4. Specific-pathogen-free (SPF) C57BL/6T wildtype mice derived bone marrow stromal cell (BMSC) assays. Untreated 10-week-old female C57BL/6T SPF mice were euthanized; BMSCs were plated for in vitro studies. BMSCs were cultured in osteogenic media to differentiate the cells into mature osteoblasts. Osteoblasts were stimulated for 14 days with no treatment control (No Tx Ctrl) or 1.25 µg/ml minocycline (MINO Tx). von Kossa assay; n=5/group: (**A**) representative images; (**B**) mineral area per well area (%). Unpaired *t*-test; reported as mean ± SEM.

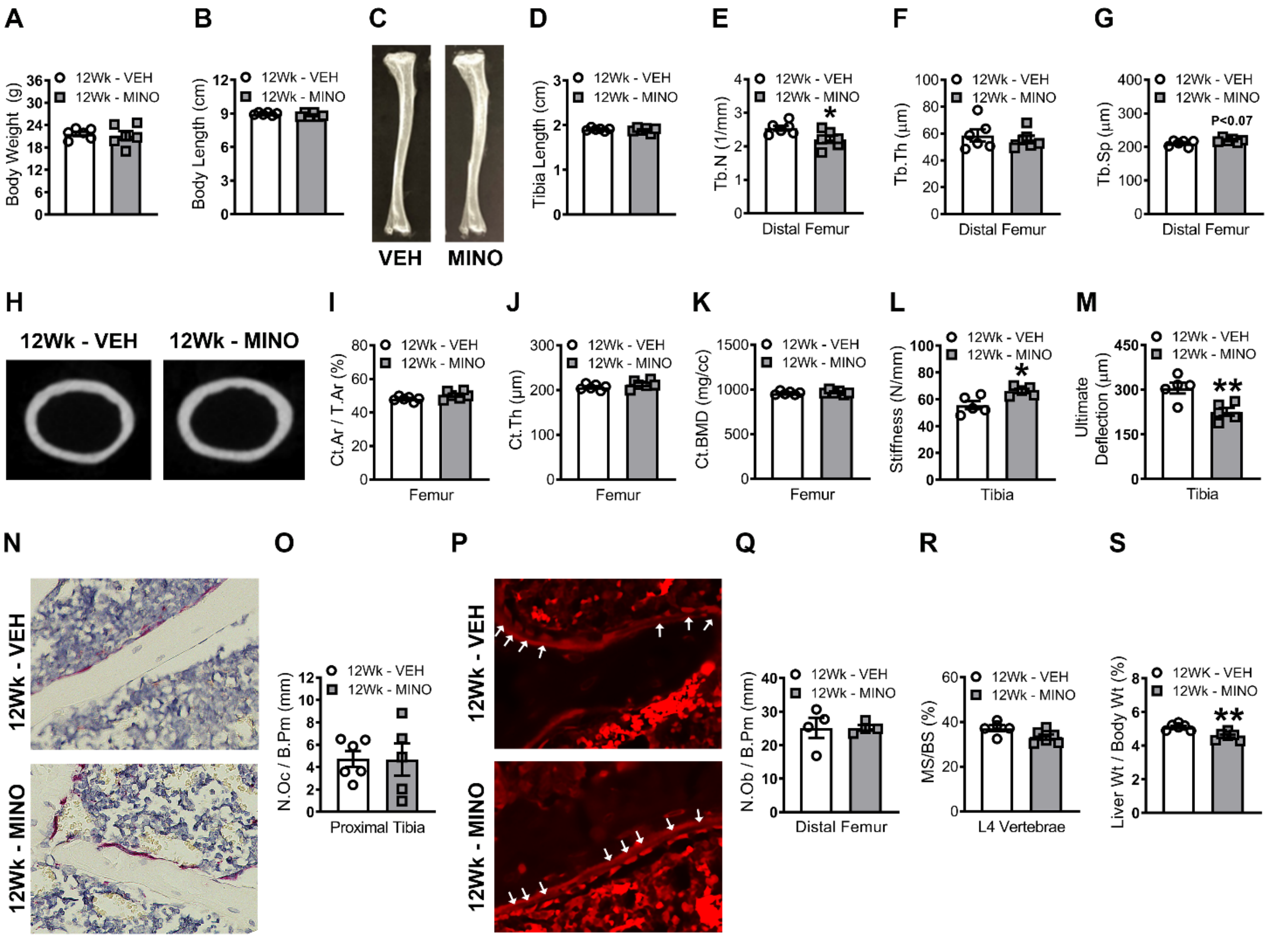

Supplemental Figure 5. Female C57BL/6T specific-pathogen-free (SPF) mice were administered vehicle-control (VEH) or minocycline (MINO) from age 6 to 12 weeks; euthanized at age 12 weeks. (**A**) Body weight; n=6/group. (**B**) Body length; n=6/group. (**C**) Representative faxitron images of tibia. (**D**) Tibia length; n=6/group. Micro-CT analysis of distal femur trabecular bone; n=6/group: (**E**) trabecular number (Tb.N); (**F**) trabecular thickness (Tb.Th); (**G**) trabecular separation (Tb.Sp). Micro-CT analysis of femur mid-diaphysis cortical bone; n=6/group: (**H**) representative images; (**I**) cortical area per tissue area (Ct.Ar/T.Ar); (**J**) cortical thickness (Ct.Th); (**K**) cortical bone mineral density (Ct.BMD). Three-point bending biomechanical analysis of tibia; n=5/group: (**L**) stiffness; (**M**) ultimate deflection. Histomorphometric analysis of tartrate-resistant acid phosphatase (TRAP)+ osteoclasts lining trabecular bone in the proximal tibia; n=5-6/group: (**N**) representative images (200x); (**O**) number of osteoclasts per bone perimeter (N.Oc/B.Pm). Immunofluorescent analysis of osteoblasts lining trabecular bone in the proximal tibia. Osterix+ cuboidal bone lining cells were designated osteoblasts (red, Osterix–Rhodamine); n=4/group: (**P**) representative images (200x), arrows indicate osteoblasts; (**Q**) number of osteoblasts per bone perimeter (N.Ob/B.Pm). Dynamic histomorphometric analysis of trabecular bone formation indices in L4 vertebra; calcein administered 5 and 2 days prior to sacrifice; n=4/group: (**R**) mineralizing surface per bone surface (MS/BS). (S) Liver weight per body weight (%); n=5-6/group. Unpaired *t*-test; reported as mean ± SEM; *p<0.05 vs. VEH, **p<0.01 vs. VEH.

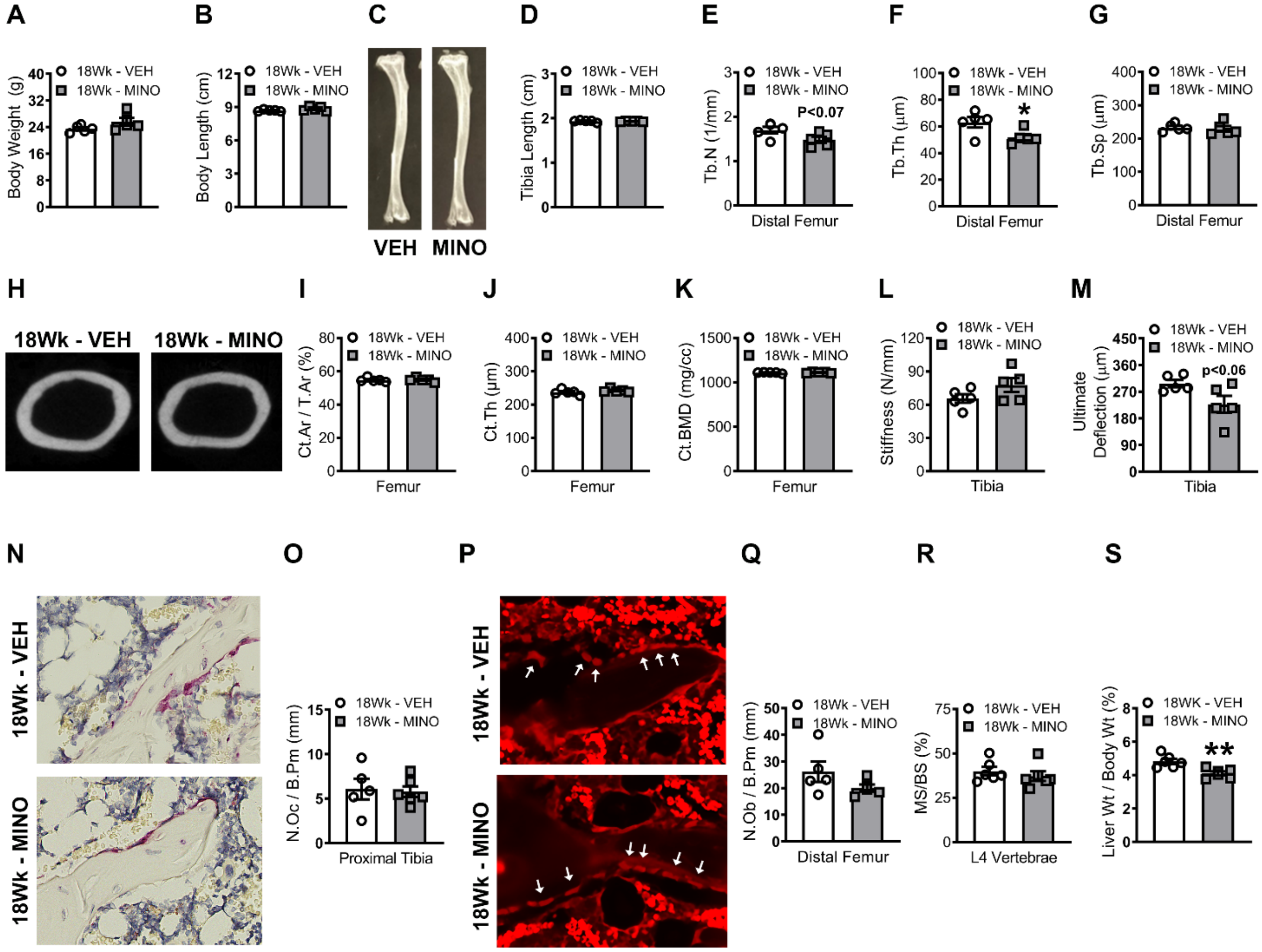

Supplemental Figure 6. Female C57BL/6T specific-pathogen-free (SPF) mice were administered vehicle-control (VEH) or minocycline (MINO) from age 6 to 12 weeks; euthanized at age 18 weeks. (**A**) Body weight; n=5/group. (**B**) Body length; n=5/group. (**C**) Representative faxitron images of tibia. (**D**) Tibia length; n=5/group. Micro-CT analysis of distal femur trabecular bone; n=5/group: (**E**) trabecular number (Tb.N); (**F**) trabecular thickness (Tb.Th); (**G**) trabecular separation (Tb.Sp). Micro-CT analysis of femur mid-diaphysis cortical bone; n=5/group: (**H**) representative images; (**I**) cortical area per tissue area (Ct.Ar/T.Ar); (**J**) cortical thickness (Ct.Th); (**K**) cortical bone mineral density (Ct.BMD). Three-point bending biomechanical analysis of tibia; n=5/group: (**L**) stiffness; (**M**) ultimate deflection. Histomorphometric analysis of tartrate-resistant acid phosphatase (TRAP)+ osteoclasts lining trabecular bone in the proximal tibia; n=5-6/group: (**N**) representative images (200x); (**O**) number of osteoclasts per bone perimeter (N.Oc/B.Pm). Immunofluorescent analysis of osteoblasts lining trabecular bone in the proximal tibia. Osterix+ cuboidal bone lining cells were designated osteoblasts (red, Osterix–Rhodamine); n=4-5/group: (**P**) representative images (200x), arrows indicate osteoblasts; (**Q**) number of osteoblasts per bone perimeter (N.Ob/B.Pm). Dynamic histomorphometric analysis of trabecular bone formation indices in L4 vertebra; calcein administered 5 and 2 days prior to sacrifice; n=5-6/group: (**R**) mineralizing surface per bone surface (MS/BS). (S) Liver weight per body weight (%); n=5/group. Unpaired *t*-test; reported as mean ± SEM; *p<0.05 vs. VEH.

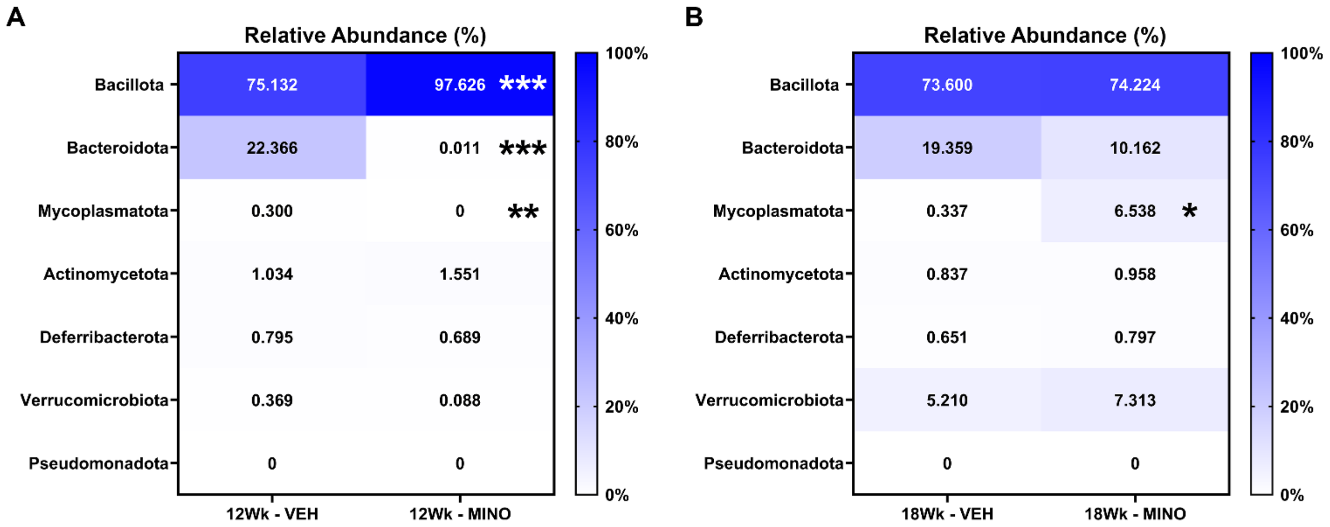

Supplemental Figure 7. Female C57BL/6T specific-pathogen-free (SPF) mice were administered vehicle-control (VEH) or minocycline (MINO) from age 6 to 12 weeks; euthanized at (**A**) age 12 weeks and (**B**) age 18 weeks. Advanced 16S rDNA sequencing analysis of the colonic bacteriome; n=5-6/group. Phyla relative abundance (%) in (**A**) 12-week-old mice and (**B**) 18-week-old mice. Unpaired *t*-test with Holm post-hoc test in 12-week-old mice and 18-week-old mice; reported as mean ± SEM; *p<0.05 vs. VEH, **p<0.01 vs. VEH, ***p<0.001 vs. VEH.

Gene	VEH	VEH	VEH	VEH	MINO	MINO	MINO	MINO	VEH Avg ± SEM	MINO Avg ± SEM	Fold Change	p-value
Nrep	26.449	16.007	21.070	10.758	51.344	69.705	68.782	32.726	18.571 ± 3.365	55.639 ± 8.728	2.996	7.43E-03
Acot3	94.069	58.382	70.762	30.689	205.798	207.977	173.861	112.525	63.476 ± 13.197	175.04 ± 22.249	2.758	5.02E-03
Nmrk1	10.316	8.250	8.652	17.994	34.439	35.993	31.714	21.899	11.303 ± 2.275	31.011 ± 3.164	2.744	2.32E-03
Tsku	25.764	42.340	32.767	156.400	242.295	145.661	129.915	176.511	64.318 ± 30.881	173.596 ± 24.86	2.699	3.30E-02
Mt1	188.659	205.198	179.296	418.124	611.323	591.480	831.003	630.913	247.819 ± 57.02	666.18 ± 55.528	2.688	1.91E-03
Pcsk4	12.173	5.299	5.047	12.350	23.829	25.455	28.808	11.910	8.718 ± 2.047	22.5 ± 3.679	2.581	1.70E-02
Cyp7a1	84.821	49.387	135.313	283.567	403.782	381.276	262.277	294.321	138.272 ± 51.54	335.414 ± 33.93	2.426	1.87E-02
Slc16a5	29.578	17.537	21.871	29.283	59.167	52.415	58.176	64.788	24.567 ± 2.944	58.636 ± 2.534	2.387	1.22E-04
Thrsp	499.662	199.022	203.492	819.249	1003.850	800.678	1393.020	852.919	430.356 ± 147.486	1012.617 ± 133.917	2.353	2.65E-02
Gadd45g	37.497	31.960	17.786	18.573	77.780	36.997	63.326	60.667	26.454 ± 4.912	59.692 ± 8.448	2.256	1.45E-02
Per2	16.035	9.779	9.694	37.484	44.915	46.257	37.730	35.555	18.248 ± 6.582	41.114 ± 2.634	2.253	1.80E-02
Nampt	64.288	56.216	39.577	82.736	153.986	117.147	106.359	139.669	60.704 ± 8.966	129.29 ± 10.766	2.130	2.72E-03
Alas1	217.453	454.975	348.657	1131.940	1245.230	1236.330	1311.980	790.715	538.256 ± 203.768	1146.064 ± 119.646	2.129	4.22E-02
Mt2	44.488	94.343	93.330	269.416	213.689	227.404	305.914	320.196	125.394 ± 49.397	266.801 ± 27.009	2.128	4.58E-02
Camk1d	147.936	170.014	204.612	165.856	78.634	82.478	58.431	124.512	172.105 ± 11.847	86.014 ± 13.874	0.500	3.26E-03
Col4a2	28.502	28.900	28.361	21.854	13.218	11.268	11.166	17.498	26.904 ± 1.687	13.288 ± 1.481	0.494	9.11E-04
Nt5e	66.928	62.991	83.880	51.282	24.413	30.473	27.023	47.080	66.27 ± 6.745	32.247 ± 5.098	0.487	6.93E-03
Adnp	28.645	12.360	17.330	22.320	9.630	9.174	7.320	12.897	20.164 ± 3.482	9.755 ± 1.16	0.484	2.97E-02
Eps8l2	39.675	53.344	75.682	37.002	15.871	28.041	20.478	34.658	51.426 ± 8.842	24.762 ± 4.144	0.482	3.42E-02
F8	13.396	27.589	22.673	24.411	15.646	5.064	5.201	15.961	22.017 ± 3.049	10.468 ± 3.081	0.475	3.73E-02
Sh3pxd2a	1.907	1.639	0.961	1.882	1.664	0.867	0.918	1.572	1.597 ± 0.22	1.255 ± 0.21	0.786	3.05E-01
Ndrg1	21.853	34.855	49.030	22.770	12.769	14.507	12.441	20.117	32.127 ± 6.366	14.958 ± 1.778	0.466	4.08E-02
Cldn1	59.155	51.628	90.450	36.954	16.770	29.059	22.230	42.261	59.546 ± 11.285	27.58 ± 5.501	0.463	4.37E-02
Tjp3	25.569	19.121	32.526	17.174	8.452	10.173	10.147	13.447	23.598 ± 3.475	10.555 ± 1.045	0.447	1.14E-02
Sgk2	62.235	56.108	60.566	67.780	17.894	29.105	29.828	30.944	61.672 ± 2.412	26.943 ± 3.04	0.437	1.09E-04
Depp1	68.249	80.910	40.778	59.531	29.269	28.192	31.255	19.873	62.367 ± 8.429	27.147 ± 2.506	0.435	7.08E-03
Shank2	15.449	19.121	36.132	21.082	7.059	9.078	6.934	15.647	22.946 ± 4.548	9.68 ± 2.049	0.422	3.75E-02
Syne1	22.635	28.136	39.497	29.573	8.273	12.135	8.923	19.244	29.96 ± 3.513	12.144 ± 2.513	0.405	6.18E-03
Nr1d1	108.967	150.381	84.170	61.050	27.998	54.619	37.865	38.196	101.142 ± 19.107	39.669 ± 5.516	0.392	2.14E-02
Slc30a10	55.545	66.515	79.591	30.834	16.837	18.290	17.172	38.437	58.121 ± 10.339	22.684 ± 5.26	0.390	2.24E-02

Supplemental Table 1. Female C57BL/6T specific-pathogen-free (SPF) mice were administered vehicle-control (VEH) or minocycline (MINO) from age 6 to 12 weeks; euthanized at age 12 weeks. Liver RNA-seq analysis in MINO- vs. VEH-treated female SPF mice at age 12 weeks; n=4/group. Unpaired t-test; reported as mean ± SEM and fold change with $p<0.05$.

Gene	VEH	VEH	VEH	VEH	MINO	MINO	MINO	MINO	VEH Avg ± SEM	MINO Avg ± SEM	Fold Change	p-value
Acot3	139.924	90.015	42.795	95.399	279.375	302.730	182.822	307.900	92.033 ± 19.861	268.207 ± 29.13	2.914	2.46E-03
Socs2	21.636	14.906	5.725	24.327	41.671	54.561	56.085	30.479	16.649 ± 4.145	45.699 ± 6.016	2.745	7.31E-03
Col27a1	9.118	8.483	6.434	8.687	8.655	25.407	20.758	25.776	8.18 ± 0.597	20.149 ± 3.998	2.463	2.53E-02
Sik1	31.156	27.536	9.995	18.018	31.749	74.154	57.783	42.191	21.676 ± 4.779	51.469 ± 9.262	2.374	2.89E-02
Nrep	34.742	16.965	12.383	34.628	60.584	68.196	47.256	54.894	24.68 ± 5.852	57.733 ± 4.429	2.339	4.09E-03
Cyp7a1	115.030	135.941	54.103	88.480	167.620	282.228	123.405	241.176	98.388 ± 17.67	203.607 ± 35.731	2.069	3.86E-02
Egr1	34.570	18.662	30.107	50.434	17.972	14.020	21.383	8.458	33.443 ± 6.58	15.459 ± 2.776	0.462	4.54E-02
Depp1	103.621	58.204	132.402	96.847	82.706	16.371	47.588	24.639	97.769 ± 15.275	42.826 ± 14.843	0.438	4.18E-02
Sgk2	31.329	38.585	75.793	55.863	27.157	17.627	20.316	22.266	50.393 ± 9.908	21.841 ± 2.011	0.433	3.02E-02
Nr1d1	79.727	64.808	179.126	117.005	72.101	19.485	45.206	9.468	110.167 ± 25.472	36.565 ± 14.034	0.332	4.46E-02

Supplemental Table 2. Female C57BL/6T specific-pathogen-free (SPF) mice were administered vehicle-control (VEH) or minocycline (MINO) from age 6 to 12 weeks; euthanized at age 18 weeks. Liver RNA-seq analysis in MINO- vs. VEH-treated female SPF mice at age 18 weeks; n=4/group. Unpaired t-test; reported as mean ± SEM and fold change with $p<0.05$.

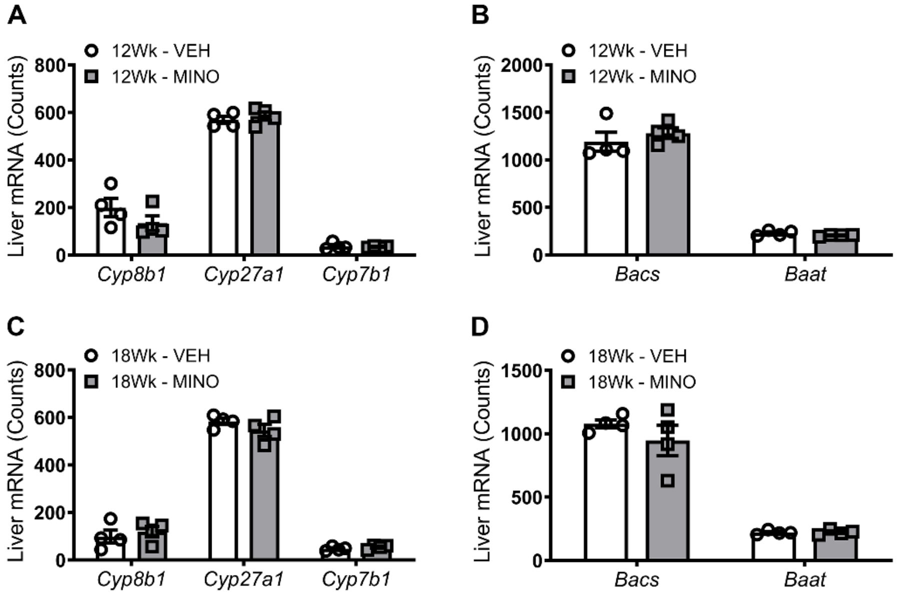

Supplemental Figure 8. Female C57BL/6T specific-pathogen-free (SPF) mice were administered vehicle-control (VEH) or minocycline (MINO) from age 6 to 12 weeks; euthanized at (**A-B**) age 12 weeks and (**C-D**) age 18 weeks; n=4/group: (**A** and **C**) mRNA counts of liver bile acid synthesis enzymes (*Cyp8b1*, *Cyp27a1*, *Cyp7b1*); (**B** and **D**) mRNA counts of liver bile acid conjugation enzymes (*Bacs*, *Baat*). Unpaired *t*-test in 12-week-old mice and 18-week-old mice; reported as mean ± SEM.

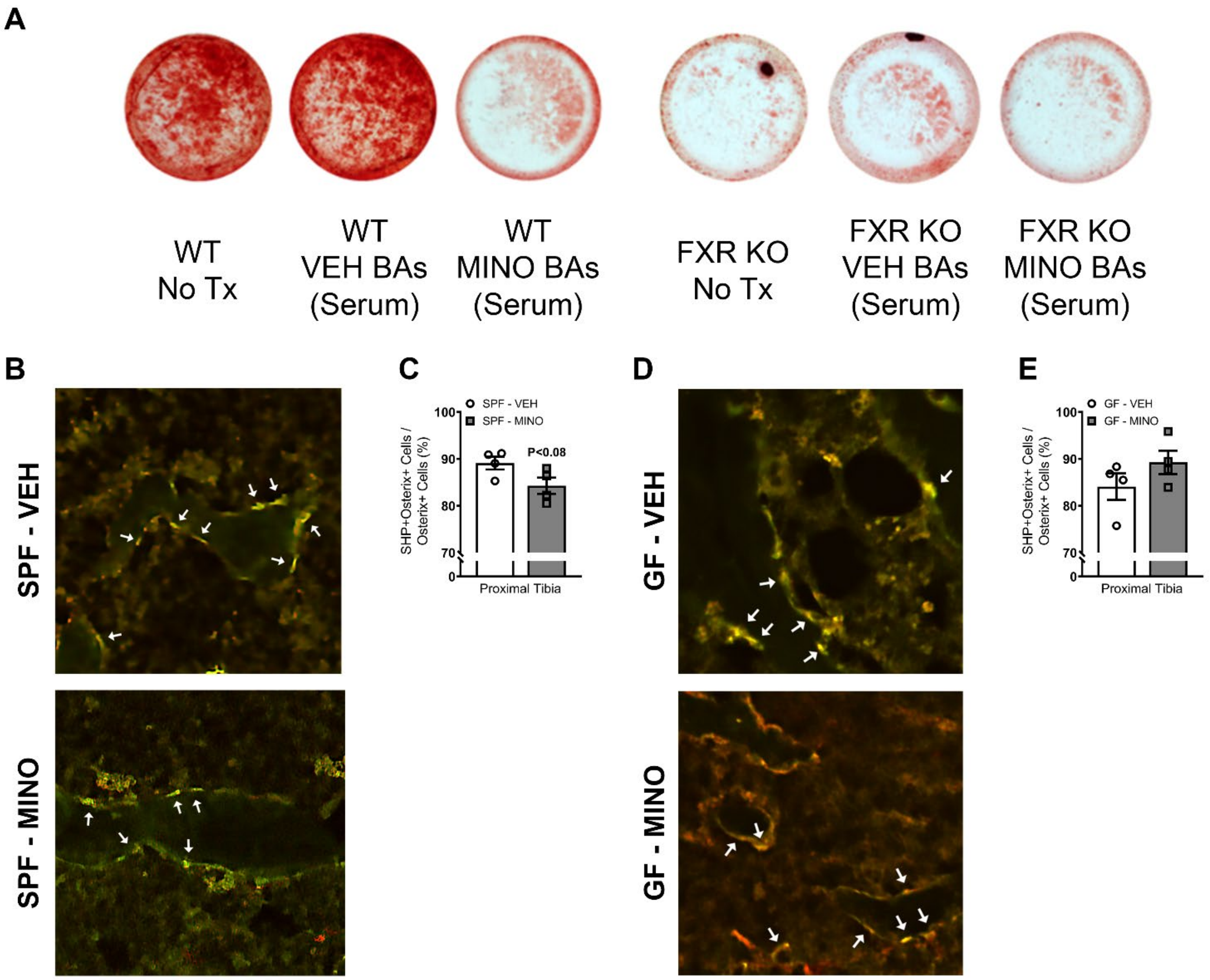

Supplemental Figure 9. (**A**) Bone marrow stromal cells (BMSCs) were isolated from untreated 10-week-old female C57BL/6J FXR knockout and wildtype mice for in vitro studies. Mature osteoblasts were stimulated with No Tx Control, MINO Serum BAs, or VEH Serum BAs; n=5/group. (**A**) alizarin red assay; representative images. Male C57BL/6T (**B** and **C**) specific-pathogen-free (SPF) and (**D** and **E**) germ-free (GF) mice were administered vehicle-control (VEH) or minocycline (MINO) from age 6 to 12 weeks; euthanized ay age 12 weeks. Immunofluorescent analysis of dual-labeled SHP+Osterix+ cuboidal osteoblasts lining bone in the distal femur (green, SHP-FITC; red, Osterix–Rhodamine); n=4/group: (**B** and **D**) representative images (100x), arrows indicate SHP+Osterix+ osteoblasts; (**C** and **E**) SHP+Osterix+ cells per Osterix+ cells (%). Unpaired *t*-test; reported as mean ± SEM.

Materials and Methods

Mice. Sex-matched five-week-old specific-pathogen-free (SPF) C57BL/6T mice were purchased from Taconic Biosciences and housed in ventilated cages in an SPF vivarium at the Medical University of South Carolina (MUSC). SPF Mice were provided one week to acclimate before initiating minocycline or vehicle-control treatment at age 6 weeks. Germ-free (GF) C57BL/6T mice were bred and housed in sterile isolators at the MUSC Gnotobiotic Animal Facility. SPF and GF mice were administered sterile-filtered 100 mg/L minocycline hydrochloride or vehicle-control drinking water from age 6 to 12 weeks. The 100 mg/L concentration drinking water supported administering a 25 mg/kg murine daily dose, which is equivalent to a 2.0 mg/kg clinical daily dose(1-3). Reports comparing antibiotic delivery modes in mice have shown that drinking water, oral gavage, and injection have similar effects on the richness and abundance of microbiota communities(4, 5). Therefore, minocycline was administered through the drinking water to limit distress and harm to the animals.

Untreated SPF mice were euthanized at age 10 weeks for in vitro studies. SPF C57BL/6T mice were purchased from Taconic Biosciences at age 8 weeks and housed in a SPF vivarium. C57BL/6J FXR knockout mice (Nr1h4tm1Gonz/J, stock number 004144) and wild-type mice were bred and housed in a SPF vivarium.

Animals were maintained on a 12hr:12hr light:dark schedule. Room temperature/humidity were maintained within the advised ranges per the NIH *Guide for the Care and Use of Laboratory Animals* (National Academies Press, 2011). SPF mice received autoclaved NIH-31M diet (Zeigler). GF mice received autoclaved Teklad 8656 diet (Harlan Laboratories).

Bacterial 16S rDNA Analyses. Genomic DNA was isolated from colonic contents using the DNeasy PowerSoil Pro Kit (Qiagen), per the manufacturer's instructions. Total DNA was quantified via Nanodrop 1000 (Thermo Fisher Scientific), per the manufacturer's instructions.

<u>Quantitative Real-Time PCR for 16S rDNA analysis:</u> Bacterial load analysis was performed via qRT-PCR amplification of the Universal 16S rDNA gene. Bacterial load was determined by normalizing the Universal 16S gene to a bacterial DNA standard (ZymoBIOMICS, Zymo Research); relative quantification performed via the $2^{-\Delta CT}$ method(6), as previously described(7, 8). Bacterial phylum-level analyses were performed via qRT-PCR amplification of 16S rDNA target genes. Bacterial phylum-level outcomes were determined by normalizing phyla

target genes to the Universal 16S gene; relative quantification performed via the $2^{-\Delta\Delta CT}$ method(9), as described previously(7, 8, 10). Assays were carried out in triplicate, technical replicate reactions. Forward (F) and reverse (R) primer sequences (Integrated DNA Technologies) included:

Universal 16S(10): F=5'-AAACTCAAAKGAATTGACGG-3'; R=5'-CTCACRRCACGAGCTGAC-3'

Pseudomonadota(10): F=5'-CIAGTGTAGAGGTGAAATT-3'; R=5'-CCCCGTCAATTCCTTTGAGTT-3'

Bacteroidota(10): F=5'-CRAACAGGATTAGATACCCT-3'; R=5'-GGTAAGGTTCCTCGCGTAT-3'

Bacillota(10): F=5'-TGAAACTYAAAGGAATTGACG-3'; R=5'-ACCATGCACCTGTC-3'

Actinomycetota(10): F=5'-TACGGCCGCAAGGCTA-3'; R=5'-TCRTCCCCACCTTCCG-3'

A 30-cycle qRT-PCR protocol was executed on the StepOnePlus system (Applied Biosystems). A 20 μl qRT-PCR reaction was prepared with 10 μl of SYBR Green Fast Master Mix (Applied Biosystems), 6.4 μl of forward/reverse primers (800 nM/μl) (Integrated DNA Technologies), and 3.6 μl of DNA (5 ng/μl). Initial denaturing step at 95°C for 5 minutes; 30 cycles at 95°C for 15 seconds, 61.5°C for 15 seconds, and 72°C for 20 seconds; final elongation step at 72°C for 5 minutes, as reported previously(7, 8, 10).

Advanced 16S rDNA sequencing analysis: Microbial DNA isolates were submitted to the North Carolina State University Genomics Sciences Laboratory for advanced 16S rDNA sequencing. V3 and V4 variable regions of bacterial 16S rDNA were amplified by PCR. Illumina sequencing adapters were added during a second PCR amplification step following cleanup. Sequenced products were collected, pooled, and sequenced on Illumina MiSeq v2 Reagent Kit (Illumina) for 2x250 cycles. Fastq files were filtered and processed using the DADA2 pipeline (version 1.21) in the R statistical programming software(11). Paired reads containing low quality scores and pathological errors were truncated. DADA2 sample inference algorithm removed sequencing errors from the data sequences(12). Paired sequences were then merged and chimera sequences were removed. SILVA release database version 132 was used to assign taxonomy of amplicon sequence variants(13). Sample data and the resulting microbial abundance table with corresponding taxonomic information were transferred to the phyloseq R statistical package (version 1.36). A minimum abundance threshold of 0.01% was applied to exclude amplicon sequence variants with low abundance. Alpha diversity was measured utilizing the Chao1 index. Relative abundances were analyzed using unpaired *t*-test with Holm post-hoc test to correct for multiple comparisons(14).

Micro-Computed Tomography (Micro-CT). Femurs and tibiae were fixed in 10% phosphate-buffered-formalin for 24 hours at room temperature and then stored in 70% ethanol at 4º. Femurs were scanned using the Skyscan 1176 (Bruker). Tibiae were scanned with Scanco Medical µCT 40 scanner (Scanco Medical).

Micro-CT images of femurs were obtained using the Skyscan 1176 (Bruker Corporation), with a 0.5 mm thick aluminum filter, and the following acquisition parameters: X-ray tube potential (peak) = 50 kVp, X-ray intensity = 497 µA, voxel size = 9 μm^3, integration time = 65 ms, and rotation step = 0.3°. Calibrated three-dimensional images were reconstructed of femurs using NRecon software (Bruker Corporation). Analyze 12.0 Bone Microarchitecture Analysis software (Analyze Direct) was utilized to analyze trabecular and cortical bone morphometry and bone mass. Distal femur trabecular bone was assessed in axial CT slices starting 350 µm proximal to the distal growth plate and extending 1800 µm (males) or 1000 µm (females) proximally; a fixed threshold of 350 Hounsfield units was utilized to identify mineralized tissue. Cortical bone was assessed in transverse CT slices in a 1000 µm segment of the femur mid-diaphysis; a fixed threshold of 500 Hounsfield units was used to delineate mineralized tissue. Analysis was performed and data is reported based on *Guidelines for Assessment of Bone Microstructure in Rodents Using Micro–Computed Tomography*(15), as previously described(16).

Micro-CT images of tibiae were obtained with the Scanco µCT 40 scanner (Scanco Medical), using the following acquisition parameters: X-ray tube potential (peak) = 70 kVp, X-ray intensity = 114 µA, integration time = 200 ms, and voxel size = 10 μm^3. Calibrated three-dimensional images were reconstructed, and Analyze 12.0 Bone Microarchitecture Analysis software (Analyze Direct) was utilized to assess trabecular and cortical bone morphometry and bone mass. Proximal tibia trabecular bone was evaluated by axial CT slices beginning 250 µm distal to the proximal growth plate and extending 1200 µm distally; a fixed threshold of 1450 Hounsfield units was utilized to identify mineralized tissue. Cortical bone was assessed by transverse slices in a 1000 µm segment of the tibia mid-diaphysis; a fixed threshold of 1600 Hounsfield units was used to delineate mineralized tissue. Analysis was performed and data is reported based on *Guidelines for Assessment of Bone Microstructure in Rodents Using Micro–Computed Tomography*(15), as previously described(17, 18).

Micro-Radiographs. Micro-radiographs of tibiae were acquired via Faxitron LX-60 (Faxtiron X-ray); exposure = 40 seconds; beam energy = 36 kVp.

Histology/Histomorphometry. Tibiae were fixed in 10% phosphate-buffered-formalin for 24 hours at room temperature and then stored in 70% ethanol at 4º. Tibiae were decalcified in 14% EDTA for 21 days at room temperature and submitted for paraffin histological processing. Five µm serial frontal sections were cut from proximal tibiae. Sections were stained with tartrate-resistant acid phosphatase (TRAP) and counterstained with hematoxylin. Histomorphometric analysis of TRAP+ cells lining the trabecular bone were scored as osteoclasts. The region of interest for analysis was the secondary spongiosa; initiated 250 µm distal to the proximal growth plate and extending 1000 µm distally; 50 µm from endocortical surfaces. Images were acquired at 200x utilizing the Keyence BZ-X810 microscope (Keyence) and scored via ImageJ software (ImageJ 1.53k; NIH). Data are reported based on guidelines for bone histomorphometry standardized nomenclature(19), as previously described(17, 18).

Vertebrae were fixed in 10% phosphate-buffered-formalin at room temperature for 24 hours and then stored in 70% ethanol at 4º. Tissues were dehydrated in xylenes and graded ethanol and processed undecalcified in modified methyl methacrylate(17, 20). 20mg/kg calcein was administered 5 and 2 days prior to sacrifice via intraperitoneal injection to measure dynamic bone formation(17, 21). Eight µm serial coronal sections were cut from the L4 vertebrae. Dynamic histomorphometric analyses of pulsed calcein labels was performed in unstained sections under ultraviolet illumination. The region of interest for analyses was the trabecular bone secondary spongiosa; initiated 250 µm from the cranial and caudal growth plates; 50 µm from endocortical surfaces. Images were acquired at 200X utilizing the Keyence BZ-X810 microscope (Keyence) and scored via ImageJ software (ImageJ 1.53k; NIH). Data are reported based on guidelines for bone histomorphometry standardized nomenclature(19), as previously described(17, 21).

Median liver lobes and right kidneys were fixed in 10% phosphate-buffered-formalin at room temperature for 24 hours and submitted for paraffin histological processing. Five µm transverse sections were cut from liver/kidney specimens and stained with H&E for histopathological evaluation by two independent pathologists. Five µm transverse sections were cut from liver specimens and stained by the periodic acid-Schiff (PAS) method.

Images were acquired at 200x utilizing the Nikon Eclipse TS1000 microscope (Nikon). Five randomly selected images were scored by two independent investigators via ImageJ software (ImageJ 1.53k; NIH).

In Situ Immunofluorescence. Femurs and tibiae were fixed in 10% phosphate-buffered-formalin for 24 hours at room temperature and then stored in 70% ethanol at 4º. Long bones were decalcified in 14% EDTA for 21 days at room temperature and submitted for paraffin histological processing. Five µm serial frontal sections were cut from proximal tibiae and five µm serial frontal sagittal sections were cut from the distal femur. Samples were deparaffinized with xylenes, rehydrated with graded ethanols, and briefly washed in 1X PBS. Antigen retrieval was performed in 0.2M boric acid at 60°C overnight. Samples were cooled to room temperature and washed in deionized water. Specimens were blocked in 10% goat serum for 30 minutes at room temperature. Specimens were then incubated with a 1:100 dilution of anti-Osterix monoclonal antibody (Santa Cruz Biotechnology) and a 1:100 dilution of anti-NR0B2 (SHP) polyclonal antibody (Invitrogen) for 2 hours at room temperature. Sections were washed in 1X PBS and then incubated with a 1:2000 rhodamine-goat anti-mouse (Santa Cruz Biotechnology) and a 1:2000 FITC-goat anti-rabbit (Santa Cruz Biotechnology) for one hour at room temperature (protected from light). Samples were washed with 1X PBS and mounted via ProLong Diamond Antifade Mountant with DAPI (Life Technologies). Analysis of Osterix+ and Osterix+SHP+ osteoblastic cells lining trabecular bone were evaluated. The region of interest for analysis was the secondary spongiosa, initiated 250 µm from the growth plate and extending 1000 µm; 50 µm from endocortical surfaces. Images were acquired at 100X utilizing the Olympus FluoView FV10i LIV (Olympus) microscope or at 200X utilizing the Keyence BZ-X810 microscope (Keyence) and scored via ImageJ software (ImageJ 1.53k; NIH).

Serum ELISA Analyses. Whole blood was collected via cardiac puncture at euthanasia. Serum was isolated and stored in aliquots at -80°C. Osteocalcin (OCN; Alfa Aesar), N-terminal propeptide of type 1 procollagen (P1NP; Immunodiagnostic Systems), carboxy-terminal collagen crosslinks type I collagen (CTX-1; Immunodiagnostic Systems), tumor necrosis factor (TNF; Quantikine; R&D Systems); insulin-like growth factor 1 (IGF-1; Quantikine; R&D Systems), thyroid-stimulating hormone (TSH; Lifespan Biosciences) were evaluated by ELISA, per manufacturers' instructions. Assays were performed in duplicate, technical replicate reactions.

Serum Chemistry Analyses: Whole blood was collected via cardiac puncture at euthanasia, and serum was isolated. VetScan Comprehensive Diagnostic Profile (Zoetis) was utilized to assess serum chemistries, per the manufacturer's instructions: calcium, phosphorus (PHOS), blood urea nitrogen (BUN), alanine aminotransferase (ALT), albumin (ALB), alkaline phosphatase (ALP), and total protein (TP).

Flow Cytometry. Spleen and mesenteric lymph node (MLN) cells were isolated, washed, and counted. Cells were then resuspended for analyses.

Live cell analyses: 100,000 cells were resuspended in 50 µL of flow cytometry buffer, treated with FcR-block (Miltenyi Biotec), and then labeled for surface markers, per the manufacturer's instructions as described previously(7, 18). Dead cells were excluded via labeling with propidium iodide viability dye (Miltenyi Biotec), per the manufacturer's instructions as previously described(7, 18). *M1 macrophages*: anti-CD11b-APC (Miltenyi Biotec; clone REA592), anti-MHC II-FITC (Miltenyi Biotec; clone REA528), anti-CD206-PE (Miltenyi Biotec; clone MR6F3), anti-CD64-APC-Vio770 (Miltenyi Biotec; clone REA286). *Conventional Dendritic cells*: anti-CD11c-PE-Vio770 (Miltenyi Biotec; clone REA754), anti-CD11b-APC (Miltenyi Biotec; clone REA593), anti-B220-VioBlue (Miltenyi Biotec; clone REA755), anti-MHC II-FITC (Miltenyi Biotec; clone REA528). *Activated T-cells*: anti-CD3-PEVio770 (Miltenyi Biotec; clone REA641), anti-CD4-VioBlue (Miltenyi Biotec; clone REA604), anti-CD8-PE (Miltenyi Biotec; clone REA601), anti-CD62L-FITC (Miltenyi Biotec; clone REA828), anti-CD69-APC (Miltenyi Biotec; clone H1.2F3). A minimum of 5,000 live gated cells were analyzed per specimen. Data was acquired by the MACSQuant System (Miltenyi Biotec), and analyses were performed via FlowJo 11.0 software (TreeStar), as previously reported(7, 18).

Fixed cell analyses: 100,000 cells were resuspended in 50 µL of flow cytometry buffer, treated with FcR-block (Miltenyi Biotec), and then labeled for surface markers, per the manufacturer's instructions as described previously(7, 18). Dead cells were excluded via labeling with eFluor 780 viability dye (eBioscience), per the manufacturer's instructions as previously described(7, 18). For intracellular staining, cells were treated with fixation/permeabilization buffer (eBioscience) and then labeled for intracellular transcription factors, as previously reported(7, 18). *T_H17 cells*: anti-CD3-APC-Vio770 (Miltenyi Biotec; clone REA641), anti-CD4-FITC (Miltenyi Biotec; clone REA604), anti-RORγt-APC (Miltenyi Biotec; clone REA278), anti-AHR-PE-Vio770 (eBioscience;

clone 4MEJJ). *T_{REG} cells*: anti-CD3-APC-Vio770 (Miltenyi Biotec; clone REA641), anti-CD4-FITC (Miltenyi Biotec; clone REA604), anti-CD25-PE-Vio770 (Miltenyi Biotec; clone 7D4), anti-FoxP3-PE (Miltenyi Biotec; clone REA788). A minimum of 5,000 live gated cells were analyzed per specimen. Data was acquired by the MACSQuant System (Miltenyi Biotec), and analyses were performed via FlowJo 11.0 software (TreeStar), as previously reported(7, 18).

Three-Point Bending Test. Tibiae weight and length were recorded. Bones were soaked in phosphate-buffered saline and stored at -20°C until use. Before mechanical testing, the tibiae were thawed at room temperature. ElectroForce 3220 system (Bose) with a three-point bending apparatus was utilized for tibia biomechanical analysis. Tibia were tested in the posterior to anterior direction until failure at a constant displacement rate of 0.025 mm/s. A 50-lb load cell (Honeywell Sensotec) was used to measure the load applied to the tibia. A linear variable differential transducer was utilized to measure mid-diaphyseal displacement. Load and deflection data were recorded using the WinTest system (version 2.0; Bose). Ultimate load and deflection for each tibia were determined from load-deflection curves. The slope of the linear region of the load-deflection curve was used to measure stiffness. The ultimate load was the maximum load attained prior to fracture and ultimate deflection was the corresponding deflection. Outcomes are reported as previously described(22).

Liver RNA sequencing (RNA-seq). Left liver lobes were flash frozen at euthanasia and pulverized. RNA was isolated utilizing the RNeasy Mini Kit (Qiagen). RNA integrity and concentration was quantified via Agilent 2100 Bioanalyzer (Agilent Technologies) and NanoDrop 1000 (Thermo Fisher Scientific), per manufacturers' instructions. RNA libraries were prepared utilizing the NEBNext Ultra II Directional RNA Library preparation kit coupled with polyA purification (New England BioLabs), per the manufacturer's instructions. Pair-end sequencing was performed with a read length of 2x150 base pairs on the Illumina NovaSeq 6000 S4 platform (Illumina) at the VANTAGE facility (Vanderbilt University Medical Center). RNA-seq analysis was carried out using the Partek Flow Software (Partek). Adapter sequences were trimmed from data and pre-alignment quality assurance/quality control (QA/QC) was performed using STAR 2.7.3a index. Reads were quantified and normalized to the Mus Musculus (mouse) – mm10 genome assembly. Data is reported as total transcript counts or fold difference.

Quantitative Real-Time PCR for mRNA Analysis. Ilea and left liver lobes were flash-frozen, pulverized, and homogenized in TRIzol Reagent (Invitrogen). BMSC/osteoblast cultures were washed twice with 1X PBS and TRIzol Reagent was directly applied to cultures. RNA was isolated by the TRIzol method, per the manufacturer's instructions(7, 16). Total RNA was quantified via NanoDrop 1000 (Thermo Fisher Scientific). cDNA was synthesized utilizing the High-Capacity cDNA Reverse Transcription Kit (Applied Biosystems), according to manufacturer's protocol(7, 16). cDNA was amplified using TaqMan Fast Advanced PCR Master Mix and TaqMan primer-probes via the StepOnePlus System (Applied Biosystems), per the manufacturer's protocol(7, 16). *Gapdh* was used as an endogenous housekeeping gene; relative quantification of mRNA performed via the $2^{-\Delta\Delta CT}$ method, as previously reported(7, 16). TaqMan primer probe sequences utilized: *Gapdh* = Mm99999915_g1; *Fxr (Nr1h4)* = Mm00436425_m1; *Shp (Nr0b2)* = Mm00442278_m1; *Cyp7a1* = Mm00484152_m1; *Akp2 (Alpl)* = Mm00475834_m1; *Ocn* (*Bglap)* = Mm03413826_mH; *Runx2* = Mm00501584_m1; *Sp7* = Mm04933803_m1. Assays were performed in duplicate reactions

Ileum FGF15 ELISA Analysis. Ilea were flash-frozen, pulverized, and homogenized in T-PER Tissue Protein Extraction Reagent (Thermo Fischer Scientific). Protein concentrations were determined using the DC Protein Assay (Bio-Rad Laboratories). 20 µg of protein was loaded per well for FGF15 ELISA (Lifespan Biosciences), carried out via manufacturer's instructions. Assay was performed in triplicate reactions.

Bile Acid Proteomic Analysis. Whole blood was collected via cardiac puncture at euthanasia. Serum was isolated and stored in aliquots at -80°. Unthawed serum isolates were delivered to the U-M Metabolomics Core (Ann Arbor, MI) for bile acid proteomics analysis. Serum isolates were dried and re-suspended by reverse phase liquid chromatography and separated by liquid chromatography-mass spectrometry. Quantitation of bile acids in serum was determined using electrospray ionization-triple quadrupole-multiple reaction monitoring (ESI-QQQ MRM) methods, as reported previously(23). Data reported as concentration or relative response. Relative response is the peak area under the curve of a compound normalized to an internal standard which is added to each specimen at a standard concentration to account for variation in instrument response, injection volume, and sample preparation. The relative response is used for relative comparisons between samples.

In vitro Bone Marrow Stromal Cell (BMSC) Assays. For each animal, bone marrow from femurs and tibiae was flushed with α-MEM media (Gibco), supplemented with 20% FBS (Hyclone) and 1% PSG (2 mM glutamine, 100 U/ml penicillin, 100 mg/ml streptomycin); cells were disassociated, counted, and plated in a 60-mm dish. Twenty-four hours after plating the whole bone marrow, non-adherent hematopoietic cells were decanted and discarded, and fresh media was added back to the cultures. Forty-eight hours later, adherent cells were isolated for BMSC assays. Adherent BMSCs were washed, trypsinized, counted, and plated in α-MEM media (Gibco), supplemented with 10% FBS (Hyclone) and 1% PSG, for downstream assays. Media was refreshed every other day. Marrow cells were not combined from animals for initial whole bone marrow cultures or subsequent BMSC cultures; n-values reported for in vitro assays represent biological replicates, as described previously(20, 21). BMSC assays were carried out in triplicate, technical replicate cultures for each biological replicate.

Minocycline Treatment Assay: BMSCs were isolated from untreated male ten-week-old C57BL/6T wild-type SPF mice. First passage BMSCs were plated at 7.5 x 10^4 cells/cm^2 in 48-well plate cultures, in α-MEM media, 10% FBS, and 1% PSG. Confluent cultures were then treated with osteogenic media (α-MEM media, 10% FBS, 1% PSG, 50 mg/ml ascorbic acid, 10 mM β-glycerophosphate) or osteogenic media supplemented with 1.25 μg/ml minocycline hydrochloride (Sigma Aldrich); media changed every other day. Following 14 days of treatment, mineralization was quantified by the von Kossa method, as reported previously(20, 21).

Osteoblast Lineage – *Fxr* Expression Analysis: BMSCs were isolated from untreated female ten-week-old C57BL/6T wild-type SPF mice. *Osteoprogenitor cell cultures:* First passage BMSCs were plated at 2.0 × 10^4 cells/cm^2 in 12-well plates, in α-MEM media, 10% FBS, 1% PSG; media changed every other day. *Osteoblast cell cultures:* First passage BMSCs were plated at 7.5 x 10^4 cells/cm^2 in 12-well plates, in osteogenic media (α-MEM media, 10% FBS, 1% PSG, 50 mg/ml ascorbic acid, and 10 mM β-glycerophosphate); media changed every other day. Following 5 days of treatment, cells were harvested for qRT-PCR gene expression analysis of *Fxr*; *Sp7* (Osterix) was used as a marker for commitment to the osteoblast lineage(24).

Bile Acid Treatment Assay - Wildtype Osteoblast Cultures: BMSCs were isolated from untreated female ten-week-old C57BL/6T wild-type SPF mice. First passage BMSCs were plated at 7.5 x 10^4 cells/cm^2 in 48-well plates for von Kossa assays and 12-well plates for gene expression assays. Cells were plated in α-MEM media,

10% FBS, and 1% PSG. Confluent cultures were then treated with osteogenic media (α-MEM media, 10% FBS, 1% PSG, 50 mg/ml ascorbic acid,10 mM β-glycerophosphate) to differentiate the cells into osteoblasts; media changed every other day. To evaluate the effects of circulating/serum bile acids on osteogenesis, osteoblast cultures were stimulated for 4 days and 10 days with no treatment control (osteogenic medial), or the altered serum bile acid profiles from 18-week-old minocycline-treated female SPF mice [osteogenic media supplemented with 133 nM TCDCA, 122 nM TUCDA, 54 nM THCA, 206 nM THDCA (Cayman Chemical)] versus 18-week-old vehicle-treated female SPF mice [osteogenic media supplemented with 89 nM TCDCA, 118 nM TUDCA, 34 nM THCA, 150 nM THDCA (Cayman Chemical)]; media changed every other day. Following 10 days of treatment, mineralization was quantified by the von Kossa method, as reported previously(20, 21). Following 5 days of treatment, cells were harvested from 12-well plates for qRT-PCR gene expression analysis of *Akp2/Alpl, Ocn/Bglap*, *Runx2*, *Sp7, Fxr*, and *Shp*(20, 21).

<u>Bile Acid Treatment Assay – FXR Knockout Osteoblast vs. Wildtype Osteoblast Cultures:</u> BMSCs were isolated from untreated female ten-week-old C57BL/6J FXR knockout and wild-type SPF mice. First passage BMSCs were plated at 7.5 x 10^4 cells/cm^2 in 48-well plate cultures, in α-MEM media, 10% FBS, and 1% PSG. Confluent cultures were then treated with osteogenic media (α-MEM media, 10% FBS, 1% PSG, 50 mg/ml ascorbic acid,10 mM β-glycerophosphate) to differentiate the cells into osteoblasts; media changed every other day. To evaluate the effects of circulating bile acid on osteogenesis, osteoblast cultures were stimulated for 14 days with no treatment control (osteogenic media), or the altered serum bile acid profiles from 18-week-old minocycline-treated female SPF mice [osteogenic media supplemented with 133 nM TCDCA, 122 nM TUCDA, 54 nM THCA, 206 nM THDCA (Cayman Chemical)] versus 18-week-old vehicle-treated female SPF mice [osteogenic media supplemented with 89 nM TCDCA, 118 nM TUDCA, 34 nM THCA, 150 nM THDCA (Cayman Chemical)]; media changed every other day. Following 14 days of treatment, mineralization was quantified by the von Kossa method(20, 21) and alizarin red method(25, 26). Alizarin red cultures were de-stained with 10% cetylpyridinium chloride and the optical density (OD) was measured at 450 nm to quantify alizarin red intensity.

References

1. Bachmanov AA, Reed DR, Beauchamp GK, and Tordoff MG. Food intake, water intake, and drinking spout side preference of 28 mouse strains. *Behav Genet.* 2002;32(6):435-43.
2. Nair AB, and Jacob S. A simple practice guide for dose conversion between animals and human. *J Basic Clin Pharm.* 2016;7(2):27-31.

3. Zaenglein AL, Pathy AL, Schlosser BJ, Alikhan A, Baldwin HE, Berson DS, et al. Guidelines of care for the management of acne vulgaris. *Journal of the American Academy of Dermatology.* 2016;74(5):945-73.e33.
4. Tirelle P, Breton J, Riou G, Déchelotte P, Coëffier M, and Ribet D. Comparison of different modes of antibiotic delivery on gut microbiota depletion efficiency and body composition in mouse. *BMC microbiology.* 2020;20(1):340.
5. Kelly SA, Nzakizwanayo J, Rodgers AM, Zhao L, Weiser R, Tekko IA, et al. Antibiotic Therapy and the Gut Microbiome: Investigating the Effect of Delivery Route on Gut Pathogens. *ACS infectious diseases.* 2021;7(5):1283-96.
6. Livak KJ, and Schmittgen TD. Analysis of relative gene expression data using real-time quantitative PCR and the 2(-Delta Delta C(T)) Method. *Methods.* 2001;25(4):402-8.
7. Swanson BA, Carson MD, Hathaway-Schrader JD, Warner AJ, Kirkpatrick JE, Corker A, et al. Antimicrobial-induced oral dysbiosis exacerbates naturally occurring alveolar bone loss. *Faseb j.* 2021;35(11):e22015.
8. Hathaway-Schrader JD, Aartun JD, Poulides NA, Kuhn MB, McCormick BE, Chew ME, et al. Commensal oral microbiota induces osteoimmunomodulatory effects separate from systemic microbiome in mice. *JCI Insight.* 2022;7(4).
9. Schmittgen TD, and Livak KJ. Analyzing real-time PCR data by the comparative C(T) method. *Nat Protoc.* 2008;3(6):1101-8.
10. Bacchetti De Gregoris T, Aldred N, Clare AS, and Burgess JG. Improvement of phylum- and class-specific primers for real-time PCR quantification of bacterial taxa. *J Microbiol Methods.* 2011;86(3):351-6.
11. Callahan B, Proctor D, Relman D, Fukuyama J, and Holmes S. REPRODUCIBLE RESEARCH WORKFLOW IN R FOR THE ANALYSIS OF PERSONALIZED HUMAN MICROBIOME DATA. *Pac Symp Biocomput.* 2016;21:183-94.
12. Callahan BJ, McMurdie PJ, Rosen MJ, Han AW, Johnson AJA, and Holmes SP. DADA2: High-resolution sample inference from Illumina amplicon data. *Nature Methods.* 2016;13(7):581-3.
13. Quast C, Pruesse E, Yilmaz P, Gerken J, Schweer T, Yarza P, et al. The SILVA ribosomal RNA gene database project: improved data processing and web-based tools. *Nucleic Acids Res.* 2013;41(Database issue):D590-D6.
14. Holm S. A Simple Sequentially Rejective Multiple Test Procedure. *Scandinavian Journal of Statistics.* 1979;6(2):65-70.
15. Bouxsein ML, Boyd SK, Christiansen BA, Guldberg RE, Jepsen KJ, and Muller R. Guidelines for assessment of bone microstructure in rodents using micro-computed tomography. *Journal of bone and mineral research : the official journal of the American Society for Bone and Mineral Research.* 2010;25(7):1468-86.
16. Hathaway-Schrader JD, Carson MD, Gerasco JE, Warner AJ, Swanson BA, Aguirre JI, et al. Commensal gut bacterium critically regulates alveolar bone homeostasis. *Laboratory investigation; a journal of technical methods and pathology.* 2021.
17. Hathaway-Schrader JD, Steinkamp HM, Chavez MB, Poulides NA, Kirkpatrick JE, Chew ME, et al. Antibiotic Perturbation of Gut Microbiota Dysregulates Osteoimmune Cross Talk in Postpubertal Skeletal Development. *Am J Pathol.* 2019;189(2):370-90.
18. Hathaway-Schrader JD, Poulides NA, Carson MD, Kirkpatrick JE, Warner AJ, Swanson BA, et al. Specific Commensal Bacterium Critically Regulates Gut Microbiota Osteoimmunomodulatory Actions During Normal Postpubertal Skeletal Growth and Maturation. *JBMR plus.* 2020;4(3):e10338.
19. Dempster DW, Compston JE, Drezner MK, Glorieux FH, Kanis JA, Malluche H, et al. Standardized nomenclature, symbols, and units for bone histomorphometry: a 2012 update of the report of the ASBMR Histomorphometry Nomenclature Committee. *Journal of bone and mineral research : the official journal of the American Society for Bone and Mineral Research.* 2013;28(1):2-17.
20. Novince CM, Michalski MN, Koh AJ, Sinder BP, Entezami P, Eber MR, et al. Proteoglycan 4: a dynamic regulator of skeletogenesis and parathyroid hormone skeletal anabolism. *J Bone Miner Res.* 2012;27(1):11-25.

21. Novince CM, Whittow CR, Aartun JD, Hathaway JD, Poulides N, Chavez MB, et al. Commensal Gut Microbiota Immunomodulatory Actions in Bone Marrow and Liver have Catabolic Effects on Skeletal Homeostasis in Health. *Sci Rep.* 2017;7(1):5747.
22. Kang IH, Baliga UK, Wu Y, Mehrotra S, Yao H, LaRue AC, et al. Hematopoietic stem cell-derived functional osteoblasts exhibit therapeutic efficacy in a murine model of osteogenesis imperfecta. *Stem Cells.* 2021;39(11):1457-77.
23. Griffiths WJ, and Sjövall J. Bile acids: analysis in biological fluids and tissues. *J Lipid Res.* 2010;51(1):23-41.
24. Novince CM, Koh AJ, Michalski MN, Marchesan JT, Wang J, Jung Y, et al. Proteoglycan 4, a novel immunomodulatory factor, regulates parathyroid hormone actions on hematopoietic cells. *The American journal of pathology.* 2011;179(5):2431-42.
25. Gregory CA, Gunn WG, Peister A, and Prockop DJ. An Alizarin red-based assay of mineralization by adherent cells in culture: comparison with cetylpyridinium chloride extraction. *Anal Biochem.* 2004;329(1):77-84.
26. Ripoll CB, and Bunnell BA. Comparative characterization of mesenchymal stem cells from eGFP transgenic and non-transgenic mice. *BMC Cell Biol.* 2009;10:3.
